# Machine Learning Enabled Photoacoustic Spectroscopy for Noninvasive Assessment of Breast Tumor Progression *In Vivo*: A Preclinical Study

**DOI:** 10.1021/acssensors.3c01085

**Published:** 2024-01-30

**Authors:** Jackson Rodrigues, Ashwini Amin, Subhash Chandra, Nitufa J. Mulla, G. Subramanya Nayak, Sharada Rai, Satadru Ray, Krishna Kishore Mahato

**Affiliations:** † Department of Biophysics, Manipal School of Life Sciences, Manipal Academy of Higher Education, Karnataka, Manipal 576104, India; ‡ Department of Computer Science and Engineering, Manipal Institute of Technology, Manipal Academy of Higher Education, Manipal 576104, India; § Department of Electronics and Communication, Manipal Institute of Technology, Manipal Academy of Higher Education, Manipal 576104, India; ∥ Department of Pathology, Kasturba Medical College Mangalore, Manipal Academy of Higher Education, Karnataka, Manipal 576104, India; ⊥ Department of Surgery, Kasturba Medical College, Manipal Academy of Higher Education, Karnataka,Manipal 576104, India

**Keywords:** athymic nude mice transport, photoacoustic probe, breast tumor xenograft, wavelet transform, support vector machine (SVM)

## Abstract

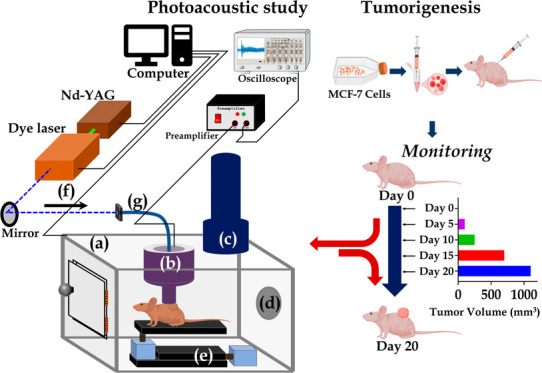

Breast cancer is a dreaded disease affecting women the most in cancer-related deaths over other cancers. However, early diagnosis of the disease can help increase survival rates. The existing breast cancer diagnosis tools do not support the early diagnosis of the disease. Therefore, there is a great need to develop early diagnostic tools for this cancer. Photoacoustic spectroscopy (PAS), being very sensitive to biochemical changes, can be relied upon for its application in detecting breast tumors *in vivo*. With this motivation, in the current study, an aseptic chamber integrated photoacoustic (PA) probe was designed and developed to monitor breast tumor progression *in vivo*, established in nude mice. The device served the dual purpose of transporting tumor-bearing animals to the laboratory from the animal house and performing PA experiments in the same chamber, maintaining sterility. In the current study, breast tumor was induced in the nude mice by MCF-7 cells injection and the corresponding PA spectra at different time points (day 0, 5, 10, 15, and 20) of tumor progression *in vivo* in the same animals. The recorded photoacoustic spectra were subsequently preprocessed, wavelet-transformed, and subjected to filter-based feature selection algorithm. The selected top 20 features, by minimum redundancy maximum relevance (mRMR) algorithm, were then used to build an input feature matrix for machine learning (ML)-based classification of the data. The performance of classification models demonstrated 100% specificity, whereas the sensitivity of 95, 100, 92.5, and 85% for the time points, day 5, 10, 15, and 20, respectively. These results suggest the potential of PA signal-based classification of breast tumor progression in a preclinical model. The PA signal contains information on the biochemical changes associated with disease progression, emphasizing its translational strength toward early disease diagnosis.

Female breast cancer is the most diagnosed cancer and the leading cause of cancer mortality worldwide. It accounts for one out of every four cancer cases and one out of every six cancer deaths and ranks first in most nations.^1^ Previous research has shown that early breast cancer identification combined with appropriate treatment might considerably lower breast cancer death rates in the long run.^2^ Currently used techniques for breast cancer diagnosis are X-ray mammography, magnetic resonance imaging (MRI), ultrasound, etc. Among them, mammography is the most popular diagnostic approach. However, this method is less sensitive for patients below the age of 40 and those with dense breast tissues and small tumors. On the other hand, MRI offers high sensitivity and specificity to subjects with dense breast tissues and can identify small breast lesions. However, the procedure is expensive and requires Gadolinium as a contrast agent, leading to central nervous system or renal damage in some instances caused by deposition. Also, MRI takes a longer diagnosis time than mammography, apart from the high cost and longer imaging duration. Ultrasound, on the other hand, despite being cost-effective compared to mammography and MRI, is radiation-free but has a high rate of false positives.^3^ Microwave imaging is another noninvasive and low-cost alternative to mammography. However, they are computationally expensive, and microwave penetration in breast tissues varies with the frequency and temperature. Therefore, developing a more practical approach suitable for detecting breast cancer noninvasively that overcomes the drawbacks of the current modalities in clinical setup is the need of the hour.^4^

Optical spectroscopy, including photoacoustic spectroscopy (PAS) in the current times, is emerging as a promising biomedical tool for investigating various disease conditions because of its sensitivity, ease of use, and simplicity supported by technological advancement in the field.^5^ However, the translational application of these tools is still in the nascent or preclinical stage and needs rigorous evaluation before reaching clinical application. There are ongoing studies on the clinical translation of fluorescence imaging in the management of breast cancer. Using label-free fluorescence imaging of cellular energy-yielding molecules and coenzymes, breast tumor regression upon immunotherapy can be monitored.^6^There are reports on real-time label-free multiphoton imaging of histological sections in diagnosing breast cancer,^7^ sentinel lymph node metastasis detection using fluorescence nanoparticles with good biocompatibility *in vitro* and *in vivo*. The potential of Near Infrared-II (NIR-II) imaging for intraoperative, early sentinel lymph node metastasis assessment has also been demonstrated.^8,9^ However, the strong optical scattering within turbid biological tissues restricts fluorescence-based imaging techniques’ imaging depth to the optical ballistic depth (<1 mm). This shallow observation depth limits fluorescence imaging techniques to superficial layers in biological tissues. On the other hand, photoacoustic spectroscopy is not influenced by the scattering losses in turbid biological media. It forms images from optically derived acoustic signals, which attenuate less than optical signals in biological tissue.^10,71^

Therefore, among optical spectroscopy tools, PAS is sensitive to detecting minor variations and/or chemical changes in biological systems. It is generated based on pulsed/modulated light excitations at specific wavelengths to a sample under study, resulting in a pressure variation due to thermoelastic expansion at the excitation volume. The signal then travels away from the excitation volume through the sample, carrying its structural/biological information/deformation along for detection by the sensor on the sample surface. The advantage of PAS in studying biological specimens is that the scattering loss of acoustic waves in tissues is several times lower than that of optical scattering, providing scopes for achieving deeper tissue information using the technique.^11,12^ Other advantages of PAS include sensitivity to optical absorption^13^ and the capacity to scan both endogenous and exogenous agents.^14^ These properties make PAS suitable for identifying and characterizing biochemical variation in a sample under study noninvasively in real time.^15^ Usually, ultraviolet (UV), visible, or infrared light sources are used to induce PA signals on biological samples targeting different biomolecules, including intrinsic proteins by UV,^16,17^ intrinsic hemoglobin/porphyrin by visible light,^18^ and extrinsic contrast agents by NIR.^19^ However, due to greater penetration by NIR compared to UV and visible light on bulk tissue specimens, it may be suitable for PA signal generation at tissue depths using targeted contrasting agents.

Numerous studies have demonstrated the potential of PAS in breast tumor diagnosis in preclinical models based on molecular/biochemical functional imaging, linking its possible application in clinical settings.^16,17^ Additionally, it can detect changes in the molecular/biochemical signatures of breast tumors, further allowing it to identify specific tumor subtypes.^20^ Also, there are reports on diagnosing breast cancer by photoacoustic and optoacoustic imaging in preclinical models. Photoacoustic-based chemical Imaging has shown its potential in radiotherapy in real time and *in vivo* mapping of tumor oxygen levels.^21^ These studies have shown that photoacoustic imaging can provide high-resolution, contrast-enhanced images of blood vessels. PAS and photoacoustic imaging are sensitive to the optical absorption properties of the biomolecules in a specimen under study and utilize photon-induced acoustic signals to characterize the samples.

In addition to breast cancer diagnosis,^22^ photoacoustic spectroscopy/imaging found its application in diagnosing and monitoring cardiovascular diseases,^23^ neurology,^24^ etc. While high-resolution images from photoacoustic-based imaging show tissue morphology very well, it has limitations in revealing detailed molecular and biochemical information.^25^ Although it promises high sensitivity and specificity, it mainly focuses on morphological features. In contrast, photoacoustic spectral data analysis (PASA) offers a deeper understanding of diseases. Unlike traditional imaging, which relies on shapes, PASA explores biomolecules using the unique biochemical and molecular properties, PASA can detect diseases early before they appear morphologically/physically. PASA brings a new perspective to medical imaging: shifting from structural details to a molecular view. Molecular/biochemical-based functional imaging is crucial for early disease detection and understanding of physiological processes. As we look ahead, integrating PASA into clinical applications can significantly enhance diagnostic capabilities, providing crucial insights into health and disease at the molecular level.^26−28^ Further, one can perform optical inversion of photoacoustic imaging by computing tissue metabolism and chromophore composition. In optical inversion, it is possible to calculate the sample’s optical properties (tissue), such as the absorption coefficient. These properties are then used to compute the concentration of different molecules in the tissue. This is called quantitative photoacoustic imaging, which is a more challenging technique than acoustic inversion, but it has the potential to provide more accurate information about the tissue. This technique is still relatively new, but it is a promising area of research when integrated with deep learning.^29^ This potential of the PAS allows molecular-specific imaging and functional analysis of the disease conditions with more insights into the changes in biochemical processes during the disease onset.^16^ However, there are no reports of photoacoustic spectroscopy-based assessment of breast tumor progression demonstrating its application *in vivo*, which uses only a single detector to capture the information compared to the imaging modality with an array of detectors. And, in this study, breast tumor progression in nude mice was assessed *in vivo* at different time points of tumor progression by a single excitation source and a single detector capturing the information.

## Materials and Methods

### Experimental Setup

**Figure 1:**
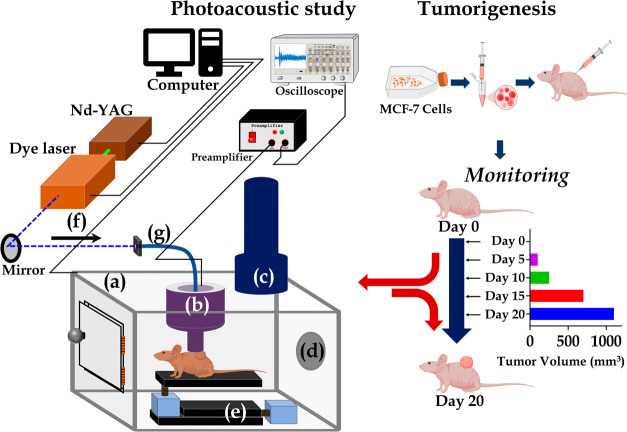
PA instrumentation setup and experimental design for the *in vivo* photoacoustic spectral study. (a) Aseptic chamber for transporting nude mice from an animal house facility in a sterile condition. (b) In-house designed and developed PA probe to excite the tumor at 281 nm through annular PZT and record the generated PA signals. (c) Air filter for providing germ-free air to the chamber during transport. (d) Exhaust, (e) translation stage to change the position using precision control, (f) pulsed laser excitation at 281 nm, and (g) optical fiber cable.

The experimental setup used for assessing breast tumor development in nude mice *in vivo* consisted of a photoacoustic probe installed in a specially designed aseptic chamber, as shown in Figure 1. The chamber designed and developed in the present study provided a sterile environment for transporting nude mice to and from the animal house facility in germ-free conditions without exposure to the external atmosphere and performing photoacoustic experiments in a laboratory *in vivo*. The sterile atmosphere of the chamber was achieved by controlling the air entering and exiting the chamber using a battery-operated air purifier, which can supply high efficienct particulate air continuously for 8 h once fully charged, and an exhaust fan releasing the inside air. The chamber was fixed with an XYZ translation stage at its bottom to remotely control the animals’ movement under study during *in vivo* photoacoustic (PA) measurements. A photoacoustic probe, consisting of a hollow core fiber for coupling laser light into the sample site under investigation, was also designed and developed in this study to record PA signals. At the delivery end of the fiber, an annular lead zirconate titanate (PZT) detector of outer diameter, 10 mm; inner diameter, 5 mm; thickness, 2 mm, placed in a Teflon casing for electrical insulation, which was further housed in an aluminum housing, was used for detecting PA signals under study. A ball lens of diameter 5 mm and focal length 2 mm was fixed at the top surface of the annular PZT in contact mode along its axis to focus the laser light onto the sample under investigation kept in contact with the PZT bottom surface. Upon 281 nm pulsed laser excitation *in vivo* at a 10 Hz repetition rate with a pulse width of 6 ns and an energy of ≈200 μJ obtained from frequency doubling of Nd:YAG second harmonic (532 nm) pumped dye laser to the samples under investigation induced PA signals in them, which the PZT of the probe then detected in physical contact with the sample using aluminum foil as a coupling medium. The experimental arrangement is illustrated in Figure 1. The complete instrumentation comprised a 281 nm pulsed laser source for excitation of the progressing tumor tissues *in vivo*, targeting tryptophan residues in proteins, a photoacoustic probe for signal detection, a preamplifier for signal amplification, and an oscilloscope for signal recording. All of the PA signals in the study were recorded for a short time (50 pulses ≈5 s/signal). As per the literature, these conditions do not cause significant damage to the biological systems/skin, and it is well within the permissible limit according to the ANSI guidelines, which allows the use of a 3 mJ/cm^2^ dose of wavelength range 180–302 nm, whereas we have used 1.02 mJ/cm^2^ dose in the current study.^16^

### Tumorigenesis

A standardized protocol was followed to induce MCF-7 cell line-derived breast tumor xenograft in nude mice (n = 5 in the test group).^16^ On day 5 post-MCF-7 cell injection, a lump at the implantation site was observed and growing further in subsequent days post-implantation. It was also observed that the tumor volume exceeded 1000 mm^3^ after day 20 postinoculation. Therefore, in the present study, the tumor was allowed to grow until day 20 post-MCF-7 cell injection, and all *in vivo* assessment of tumor progression was conducted within this period. Initially, the measurement of tumor volume was done using digital calipers at different time points of tumor progression (5, 10, 15, and 20 days post implantation), calculated using the formula [length × (width^2^)]/2 and plotted to determine tumor volume kinetics. The tumor volumes were measured for all of the animals as they reached the set time points, and the mean ± SD, *n* = 5, was calculated. The mean tumor volumes for all time points under study were analyzed by using a one-way analysis of variance (ANOVA). The animals were sacrificed upon reaching the final time point (day 20), and the tumor tissues were collected. A minute portion of the tumors were fixed using formaldehyde for the histological examination using standard protocol.^30^ Subsequently, PAS-based *in vivo* tumor progression was evaluated for all of the animals under the study at different time points of tumor growth. Another set of animals (*n* = 5) were injected only with 100% Matrigel to serve as age-matched controls without tumors.

### Assessing Breast Tumor Progression by *In Vivo* Photoacoustic Measurements

To conduct an *in vivo* PAS study, the aseptic chamber was transported to the nude mice facility, surface-sterilized using 70% ethanol, and placed inside the biosafety chamber. The animals under study were anesthetized using 50 mg/kg sodium thiopentone intraperitoneally and placed inside the chamber over the XYZ translation stage. They were then transported to the laboratory for photoacoustic spectral recordings from tumor-bearing animals *in vivo*. Once the chamber was brought to the laboratory, the external electrical connections for remote control of the XYZ translation stage were fixed. The pulsed laser light (281 nm) was coupled to the hollow core fiber to excite the tumor locations and generate the corresponding photoacoustic signals for detection. The detected signals were further amplified using a preamplifier, and the resulting output was recorded on an oscilloscope for further analysis.

For recording photoacoustic signals, the tumor location of the anesthetized animal on XYZ translation stage kept facing toward the PZT detector for precision controlled-targeted excitation. The photoacoustic signals induced in tumor tissues upon pulsed laser excitation were detected by the PZT, which, upon further amplification, were recorded on an oscilloscope in the time domain with a preset sampling frequency of 2.6 MHz. While recording the PA signals, the tumor site was adjusted for aligning with the PA probe head using X and Y movements of the translation stage. Once the alignment was achieved, the tumor region in the animal was brought in contact with the PA probe with the help of the Z motion of the stage with X & Y movements locked. In order to avoid any injury to the experimental animal, the Z stage was elevated in 100 μm increments to safely bring to the excitation site of interest in the live animal. During these adjustments, the PZT detector experiences nonspecific pressure variations caused by the movement of the animal under the influence of the XYZ translational stage movements. Hence, all recordings were conducted when the background stabilized after a while. After background stabilization, the laser was allowed to excite the targeted tumor site, and 5 spectra (5 s for each spectral recording) at the site were recorded (the PA signal obtained using the PA probe is specific to the absorbing molecule, which is shown in Figure S1). Once the PA signal recording was completed, the laser excitation was blocked, the PZT-tumor tissue contact was released, and the animal vitality was assured visually. The probe head was then fixed to different tumor sites (4 sites per tumor-bearing animal) using the movement of the XYZ translation stage following the same steps as mentioned above, and 5 corresponding PA signals were recorded. After the recording was complete, the translation stages were retracted to their respective home limits, and all of the electrical/optical connections and the chamber were transported to the animal house facility. This procedure was repeated for every individual animal under the study. In the current study, 20 spectra (5 spectra x 4 sites = 20 spectra) per animal were recorded, and a total of 100 spectra (5 spectra per site X 4 positions X 5 animals = 100 spectra) per time point was recorded and thus 500 PA spectra for all time points (day 0, 5, 10, 15, and 20) were recorded. The same procedure was followed for recording the PA signal from the age-matched control group *in vivo*. These PA spectra were subjected to data analysis using MATLAB 2019b software.

### Data Analysis

The raw, time-domain PA spectral data were stored in the oscilloscope in .txt format. These data were subsequently subjected to preprocessing, feature selection, and machine learning-based classification steps using MATLAB 2019b software, as explained in the subsequent subsections. The overall data analysis workflow is depicted in Figure S2 under Supporting Information section 3.

#### Preprocessing

The raw time-domain PA spectra and the background PA spectra of 2 ms were initially detrended. Subsequently, the PA spectra were background-subtracted. These background-subtracted spectra were subjected to normalization followed by selection of a common region of interest (ROI) with a maximum variation between 0.275 and 0.32 ms. The ROI spectral signals were subjected to wavelet transformation for further analysis. The detailed preprocessing steps for all of the time points under study (days 0, 5, 10, 15, and 20) are shown in Figures S3–S7 in the Supporting Information.

#### Wavelet Transform Analysis

Wavelet transform (WT) is a powerful mathematical tool for analyzing nonstationary signals, including time-domain photoacoustic signals. WT provides a high-frequency resolution for low-frequency components and a good time resolution for high-frequency components. Continuous wavelet transform (CWT) is more consistent and efficient due to its ability to localize time-frequency information without down-sampling.^31^ In the current study, CWT was used to transform the time-domain signals into time-frequency domain signals using the “bior 2.6” mother wavelet. The selection criteria for bior 2.6 are shown in Supporting Information Section 3 and Figure S8. The method to obtain the continuous wavelet transform (CWT) involves using an analytic Morse wavelet with symmetry parameters. The minimum and maximum scales are determined automatically by analyzing the energy spread of the wavelet in frequency and time.

#### Feature Selection

The mRMR, a filter-based feature selection algorithm, was used to select features based on the feature ranking. The feature selection algorithm aims to improve classification performance by selecting the most relevant features while eliminating noise. This process enables better classification between classes.^32^ The mRMR ranks each feature based on its mutual information to measure redundancy and relevance in the algorithm. In the study presented here, we used the mRMR to eliminate unnecessary features that might interfere with the best performance of the machine learning model.

#### Classification

The preprocessed photoacoustic spectra under study belonging to different time points were subjected to CWT analysis. The top 20 features from each spectrum were selected based on the incremental feature selection method and used as input for training and testing the support vector machine (SVM) learning models. In the current study, multiclass SVM was applied to classify the tumor tissues at different time points under study. The model was trained using 60% of the data and tested using 40% of the data, and the predicted classes were based on score values obtained through the SVM. SVM achieves class separation by creating hyperplanes through support vectors and margins. The margin represents the maximum distance between the hyperplanes of different classes (time points in this case). The kernel functions: ’SVM-RBF (Radial Basis function)’ and ’SVM-Polynomial,’ used for nonlinear data analysis, and ’SVM-Linear’^33^ used for linear data analysis was used in the current study. Among the three kernel functions, the kernel function with the best model performance in SVM analysis was finalized and further subjected to cross-validation analysis.

### Cross-Validation

In the current study, the best SVM model with the best suitable kernel function was subjected to *k*-fold cross-validation (*k* = 10) analysis. The *k* (10) folds means it consists of the data sets in 10 parts, and for testing, it uses one part, in a one-in and one-out manner, with the remaining 9 parts for training the model. The advantage of *k*-fold cross-validation is that it calculates the error performed in each step and thereby determines the overfitting of the trained SVM model.

## Results

### Tumorigenesis

**Figure 2:**
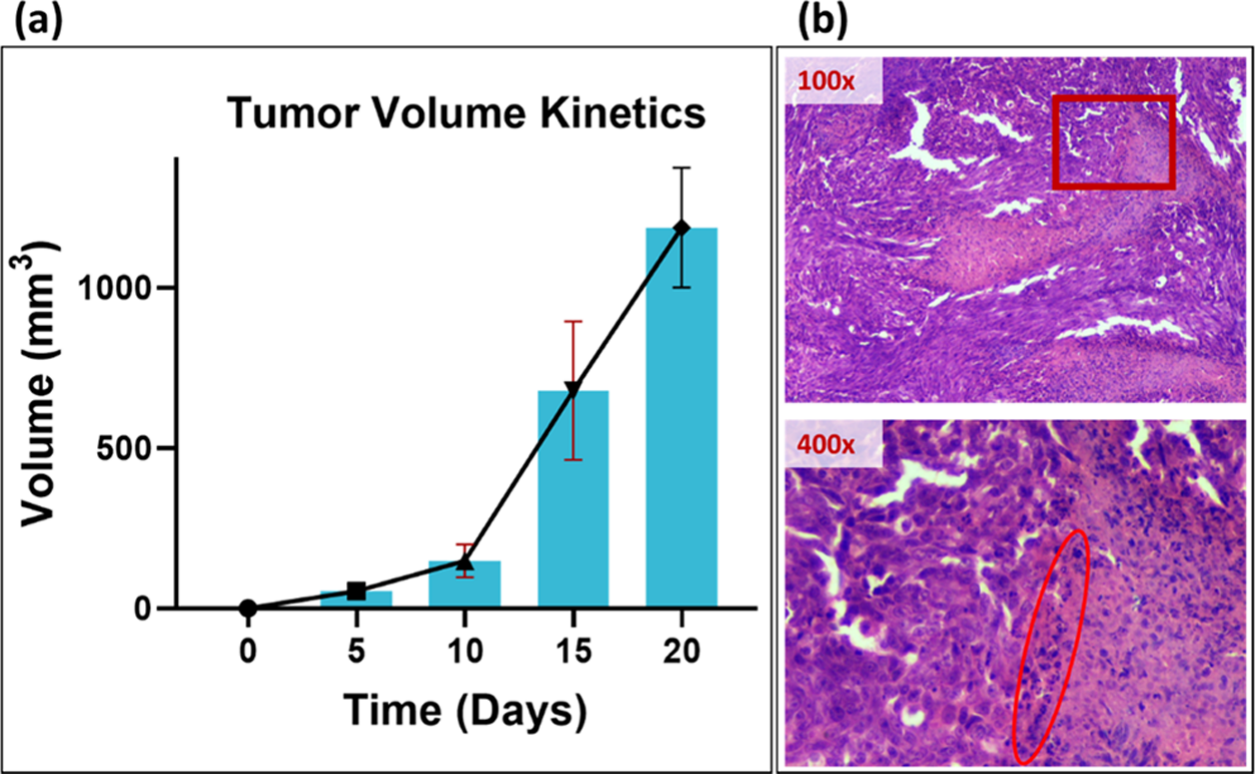
(a) Tumor volume kinetics with respect to different time points (days 0, 5, 10, 15, and 20). (b) H&E staining of Day 20 tumor tissue in 100× magnification shows necrotic and tumor cells in the top panel, and the magnified region is marked in the red box. The bottom panel shows the 400× magnification of the section showing pleomorphic nuclei and nuclear debris (DNA) in the necrotic region.

After MCF-7 cell injection, the animals were monitored for various time points of tumor development. The tumor volume kinetics was plotted as shown in Figure 2a. The results showed palpable tumor growth, starting on day 5 and progressing in subsequent days. On day 20, the tumor volume crossed 1000 mm^3^, as shown in Figure 2a. Upon performing one-way ANOVA for tumor growth at all of the time points, it was observed that the *p*-value was <0.001 (***). The outcome depicted a high significance of the increase in tumor volume, demonstrating breast tumor progression. After the *in vivo* photoacoustic spectral acquisition at different time points under study, the tumor tissues were harvested on day 20 and subjected to a histological study. The photomicrographs obtained from hematoxylin and eosin (H&E) stained sections are shown in Figure 2b in 100× and 400×. The results revealed pleomorphic and abnormal nuclei with necrosis corresponding to high-grade tumors.

### Assessing Breast Tumor Progression by *In Vivo* Photoacoustic Measurements

**Figure 3:**
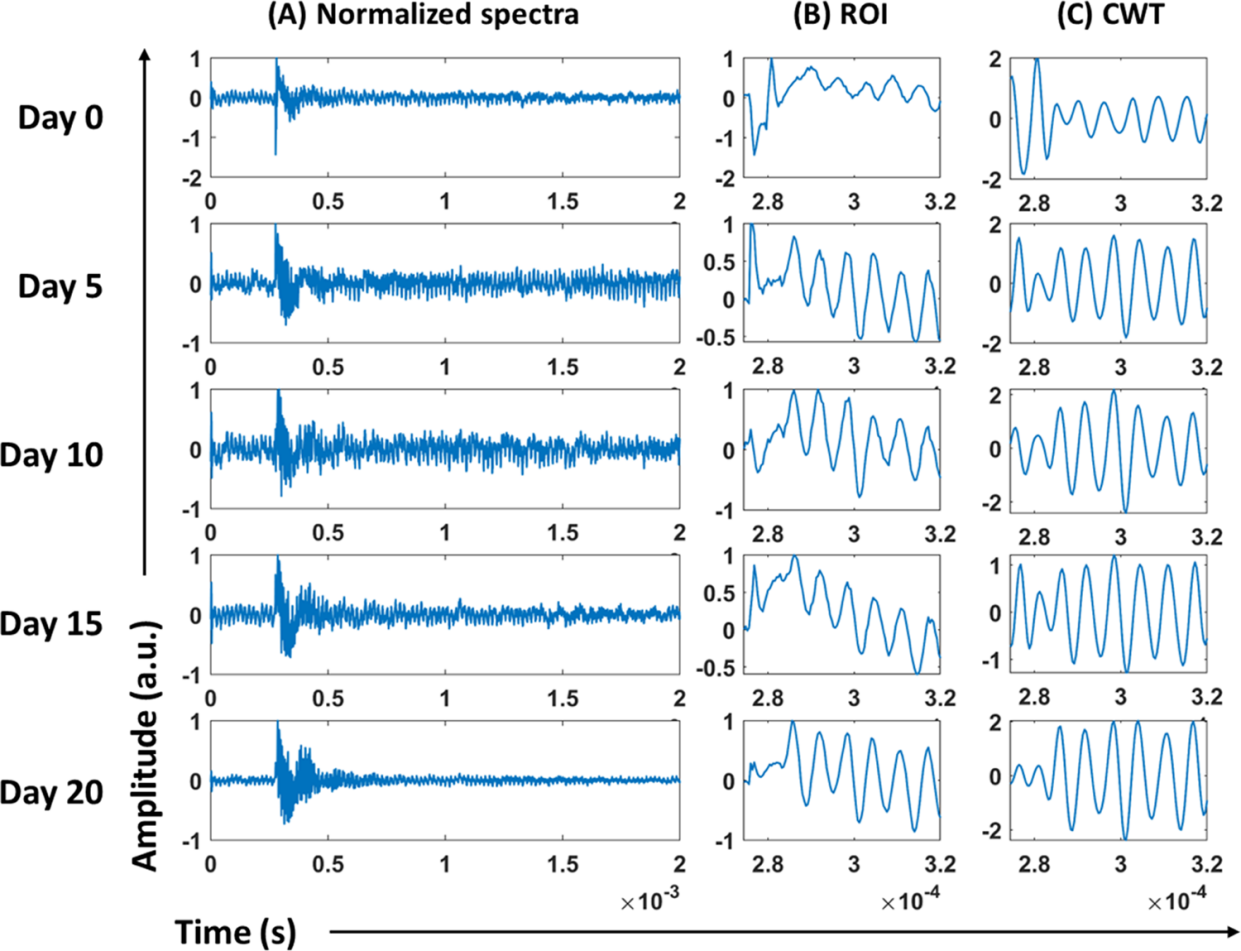
Typical preprocessed photoacoustic spectra in the time domain (0–2 ms) for different time points (Day 0, Day 5, Day 10, Day 15, and Day 20) of tumor progression *in vivo* (A), the corresponding region of interest (ROI, 0.275–0.32 ms) selected spectra (B), and continuous wavelet-transformed spectra of the ROI (C).

The typical photoacoustic spectra recorded *in vivo* in the time domain are shown in Figure 3. The current study considers the PA spectra between 0.275 and 0.32 ms of the time domain as the ROI for continuous wavelet transformation and feature selection. The feature ranking was achieved based on the mRMR algorithm applied to the wavelet-transformed ROI data under study.

### Feature Selection

**Figure 4:**
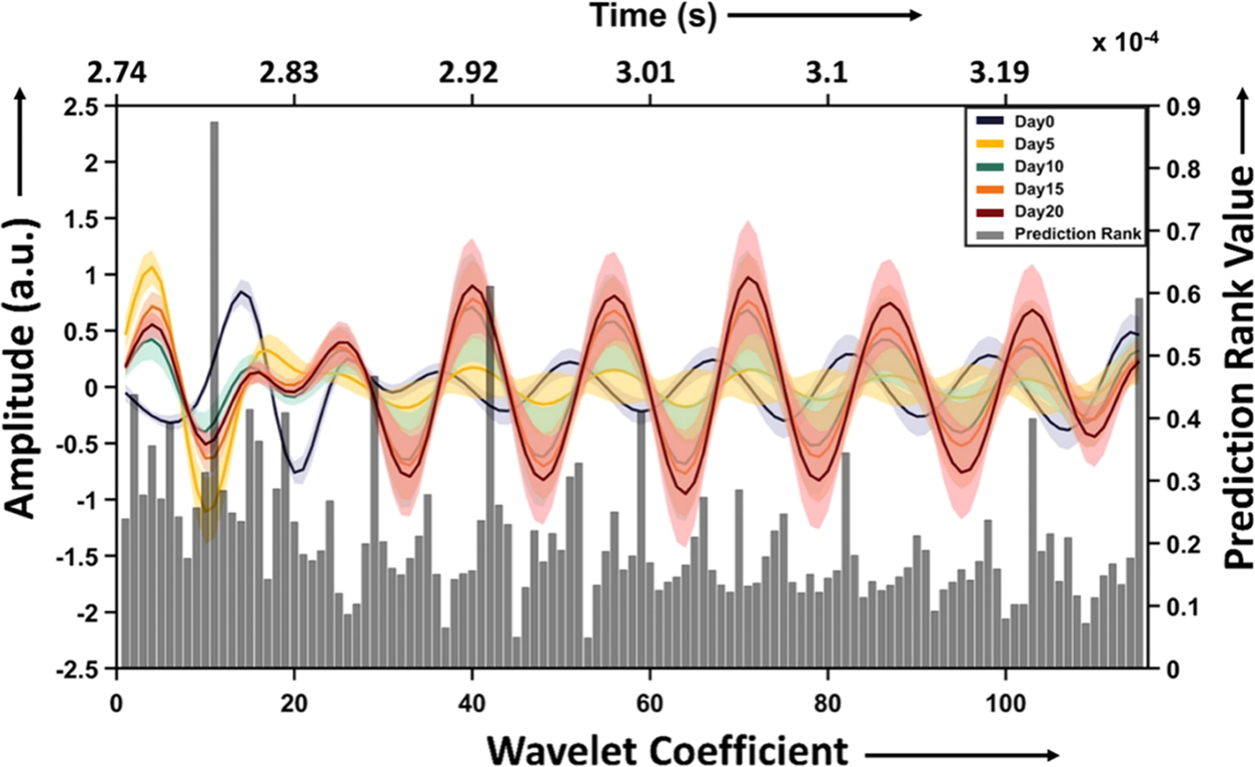
Mean photoacoustic spectra in the ROI (0.275–0.32 ms) from day 0, 5, 10, 15, and 20, after CWT (line graph)—a plot of time versus amplitude graph. The bar graph represents the features after mRMR on the input data—a plot of wavelet coefficient versus prediction rank values.

**Figure 5:**
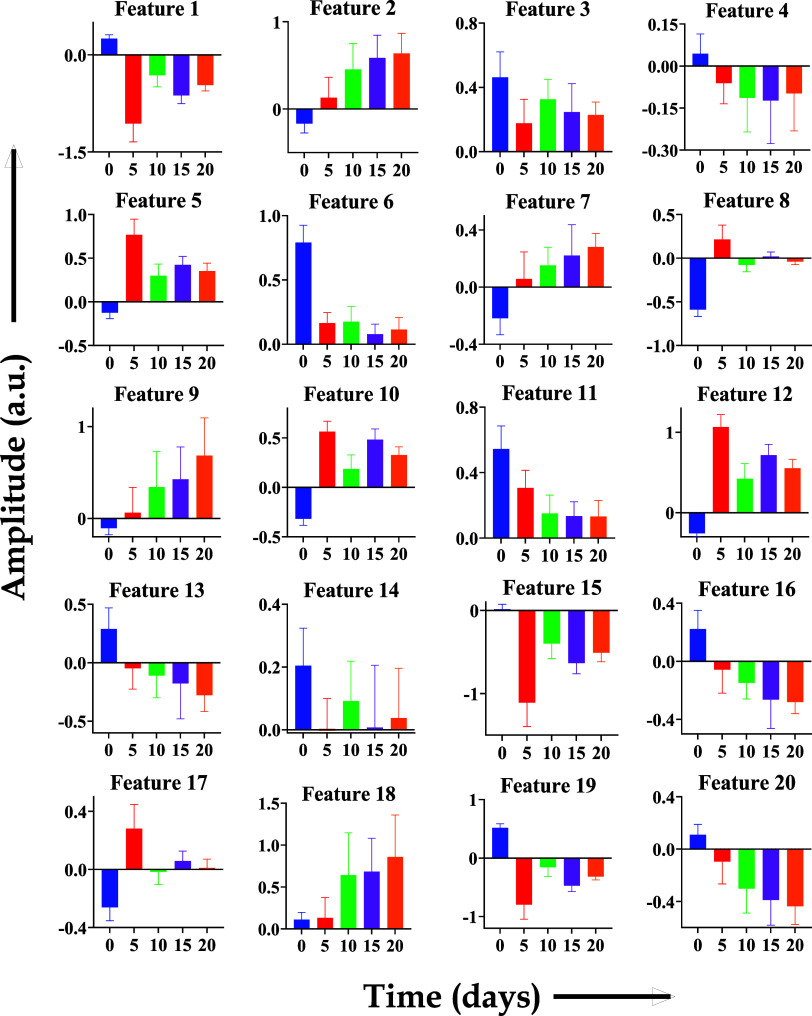
Selected top 20 features for each time point (day 0, 5, 10, 15, and 20) under study with maximum prediction ranking values.

The mean photoacoustic spectra of different experimental time points showed different CWT patterns with amplitudes varied along the time axis. The wavelet-transformed spectra were then subjected to the mRMR algorithm to select the top 20 features having maximum variations among different time points spectra under study, ranked based on their prediction rank values (PRVs), as shown in Figure 4 and Table 1. The top feature (feature 1) gave the highest prediction rank value of around 0.87, and the least PRV of the top 20 features (feature 20) was around 0.27. The details of the top 20 features selected for classification are depicted in Figure 5.

**Table 1:** List of Top 20 Features with Their Coefficients and Their Corresponding Prediction Rank Values

**sl. no.**	**coefficient**	**prediction rank value**	**sl. no.**	**coefficient**	**prediction rank value**
1	11	0.873878711755030	11	16	0.363419287399688
2	42	0.610829774390094	12	4	0.356394918613475
3	115	0.591614639895754	13	82	0.344771388857134
4	29	0.466717891698191	14	52	0.328240975164707
5	2	0.437944172381114	15	10	0.313315873973717
6	15	0.414198425087539	16	51	0.306319141534676
7	59	0.409271507019886	17	18	0.286743264810164
8	19	0.409188208614947	18	70	0.285413914246367
9	103	0.399224022616676	19	12	0.284375443939519
10	6	0.391921947316264	20	35	0.278261364763124

The top 20 selected features can be visualized, as shown in Figure 5, with differential amplitudes for each spectrum corresponding to the feature ranked by the mRMR algorithm. The amplitude of the top 20 features for the time points under study, day 0, 5, 10, 15, and 20, demonstrated variations in amplitudes. The amplitude for features 2, 7, 9, 11, 13, 18, and 20 showed linearity (increase/decrease), while in other cases, it varied in different patterns. However, upon performing one-way ANOVA, a *p*-value <0.001 was obtained. These selected features were subsequently given as input for the SVM machine learning model.

### Classification

**Figure 6:**
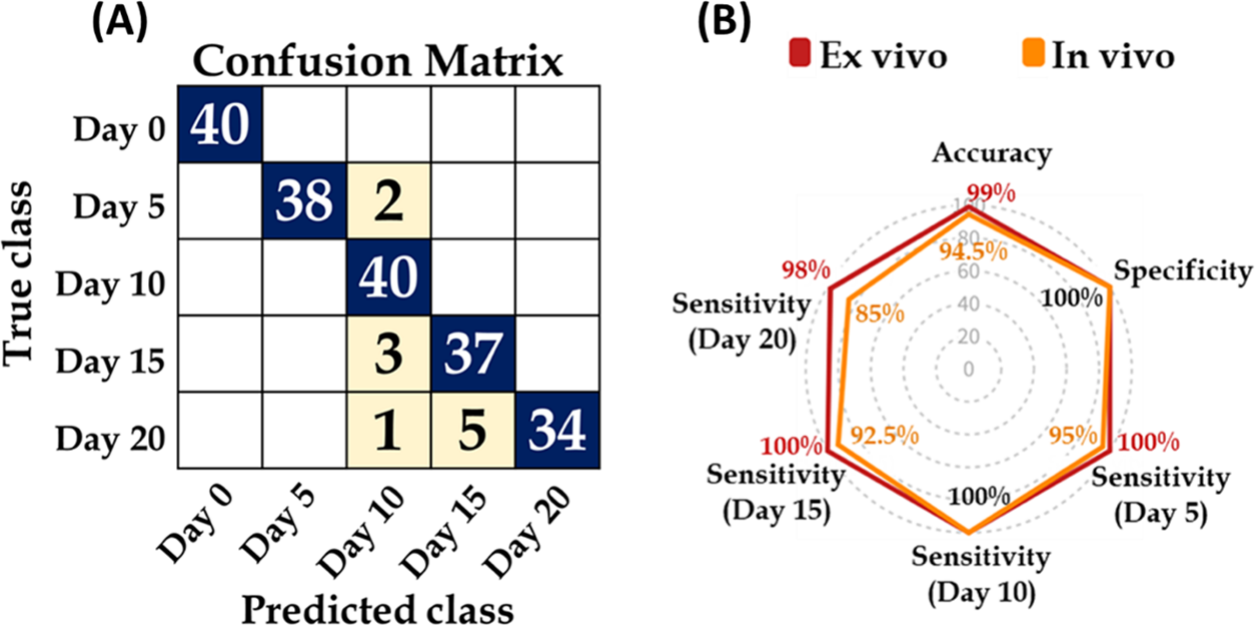
(A) Confusion matrix of SVM model after testing using 40% of the data for each time point (day 0, 5, 10, 15, and 20) under study using SVM-RBF demonstrating classification accuracy of 94.5%. (B) Spider graph showing the difference between performances for *in vivo (Orange connector lines*—*current study)* and *ex vivo (red connector lines*—*previous study)*^17^ models trained and tested using 60 and 40% data, respectively.

The top 20 features with varying amplitudes were used to train the SVM model for classification, which showed an overall accuracy of 94.5%, specificity of 100%, and sensitivity of 95, 100, 92.5, and 85% for days 5, 10, 15, and 20, respectively, as shown in Figure 6A. These results were also compared with an *ex vivo* study of similar time points previously conducted by Rodrigues et al., 2021. The *ex vivo* study reported an accuracy of 99%, specificity of 100%, and sensitivity of 100, 100, 100, and 98% for the time points, days 5, 10, 15, and 20, respectively, post-tumor induction. The performance of the *ex vivo* study was observed to be better than that of the current *in vivo* study, as shown in Figure 6B.

### Cross-Validation

**Figure 7:**
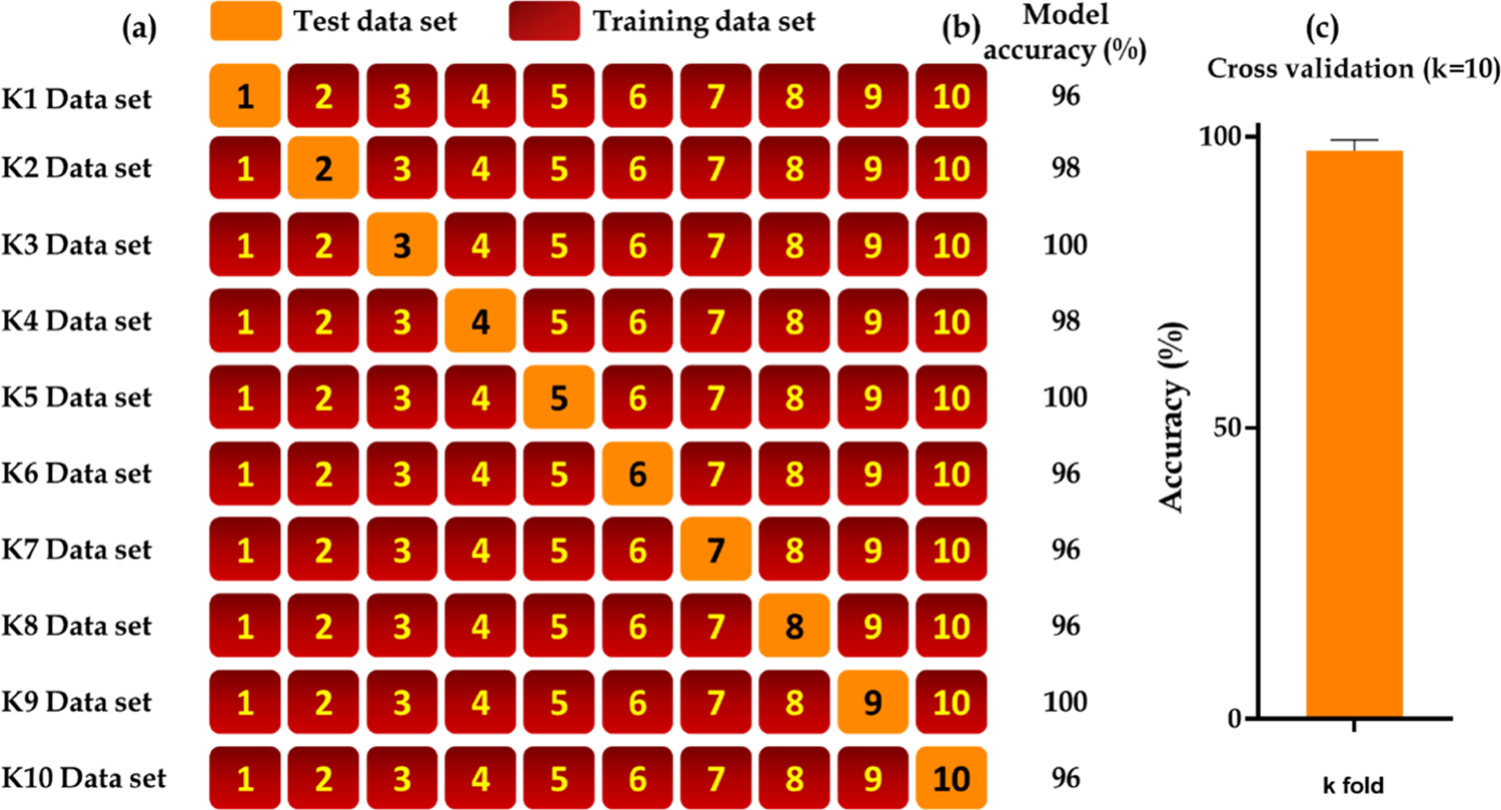
*k*-Fold cross-validation (*k* = 10) performed on the trained SVM-RBF model (using 60% training data set and 40% test data set). (a) Schematic representation of *k* data sets and (b) their corresponding accuracies for each *k*-fold. (c) Represents the mean (97.5 ± 1.75) of all *k* (10) values obtained from the *k*-fold cross-validation.

Further, in the current study, the trained machine learning model was subjected to *k*-fold cross-validation (*k* = 10). The cross-validation scheme (*k* = 10) is shown in Figure 7A, and the corresponding accuracies in each cluster (*k*) are shown in Figure 7B. The SVM-RBF model’s mean accuracy was 97.5 ± 1.75, as shown in Figure 7C.

### Age-Matched *In Vivo* Comparison of Tumor-Bearing Animals with Healthy Mice

**Figure 8:**
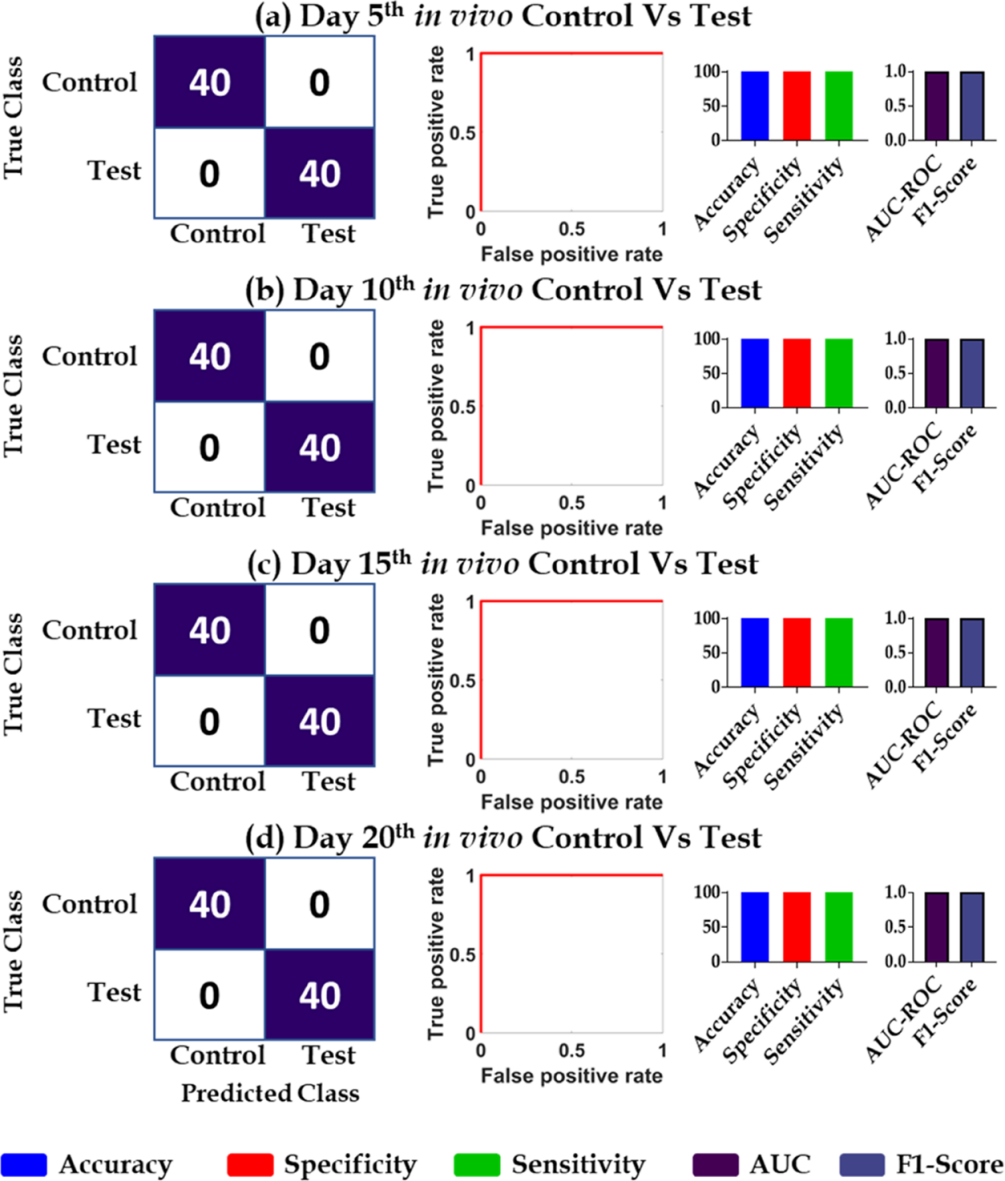
Binary classification of PA spectra from tumor-bearing animals on day 5, 10, 15, and 20, along with their respective age-matched control animals recorded *in vivo* (a–d). Representing the confusion matrix after using the test data set (40%) on the trained model using the training data set (60%), on the left panel; ROC for the model in the middle panel and the performance metrics of the trained SVM learning model represented by a bar graph on the right panel.

The breast tumor progression *in vivo* was assessed in tumor-bearing animals. There was an age-matched control group (*n* = 5), and their *in vivo* photoacoustic spectra were recorded at the site on the flank region of the mice where 100 μL of 100% Matrigel alone was injected. These animals were also monitored for 20 days, and the corresponding PA spectra were recorded at the same time points (days 0, 5, 10, 15, and 20) of the experimental group. This data was compared with the corresponding PA spectra of tumor-bearing animals. SVM-RBF was used for the binary classification, and the performance evaluation was depicted using a confusion matrix (with accuracy, specificity, and sensitivity of 100%), receiver operator characteristic (area under the curve, 1), and F1 score (1) for all of the comparisons of day 5 test Vs day 5 control, day 10 test Vs day 10 control, day 15 test Vs day 15 control, and day 20 test Vs day 20 control, as shown in Figure 8.

## Discussion

Early diagnosis of breast cancer is the best possible option to improve the patient survival rate. In early diagnosis, information about the disease is captured much before its physical and morphological appearance at the onset when the biochemical changes initiate the disease. Hence, under such conditions, there is ample scope to revert to normalcy from the disease.^34^ The spectroscopy techniques, including PAS, can capture such early biochemical changes regarding their spectral variations and conformational alterations linking them to disease conditions. Hence, they may be suitable for diagnosing diseases at early stages.^16,17,35,36^ The current study thus designed a device to demonstrate the potentiality of PAS in diagnosing progressive breast tumor conditions *in vivo* in a preclinical model without the help of any external fluorophores/chemical agents.^37^ The tumor volume kinetics obtained from the study extending for 20 days post-MCF-7 cell injection demonstrated the establishment of breast tumors in nude mice. The tumor growth was also determined by PAS during this time frame at different time points *in vivo* on the same animals.

Under the current study, an aseptic chamber was designed and developed with a built-in PA probe to measure the PA signals *in vivo* and monitor the progression of the tumor. The photoacoustic signatures were monitored in the same animals over 20 days at different time points (0, 5, 10, 15, and 20 days post-MCF-7 cells injection). The varied PA signatures and the corresponding preprocessed signals, with the tumor progression measured in the same animals over 20 days at different time points, as shown in Figure 3A–C, demonstrated the technique’s capability in detecting and monitoring tumor growth. The findings suggest that this approach is well suited for assessing the dynamic changes associated with tumor development.

The advantage of *in vivo* tumor assessment by PAS over *ex vivo* is its continuity in capturing signals from the same animals during the progression of the tumor, unlike the discontinuity in signal capturing from different animals in *ex vivo* studies. Further, monitoring breast tumor progression in the same animal consistently improves the longitudinal assessment. *In vivo*, studies track tumor size and behavior changes over time and can overcome the heterogeneity raised due to subject-to-subject variation.

The current study used an annular piezoelectric transducer in sensing and actuation, as discussed in the Materials and Methods section. One of the most widely used piezoelectric materials is PZT ceramic.^38,39^ This material is known for its rugged construction, small size, excellent frequency response, signal output with a negligible phase shift, and ability to be tailored to meet the requirements of a specific purpose. In addition, it is physically strong, chemically inert, and relatively inexpensive to manufacture. Further, PZT ceramics can be made to have greater sensitivity at higher operating temperatures than other piezoelectric ceramics, making them an ideal choice for application in PAS and imaging^40^ at absorption coefficients down to 10^–5^/cm.^41^ Piezoelectric micromachined ultrasonic transducers are also used for detecting PA signals^42,43^ in imaging and endoscopic applications.^44^

The use of 281 nm excitation wavelength in the current study was based on the ANSI guidelines for the safe use of radiation, which do not cause any damage to the biological systems with the dose of 1.02 mJ/cm^2^, as compared to the allowed limit of 3 mJ/cm^2^ dose.^16^ Further, in the current study, proteins are targeted for PA signal generation, and the 281 nm wavelength is a suitable choice for this selection. Although this radiation is harmful in a clinical scenario, seeing the severity of the disease and the maximum exposure of nearly 5 s per signal recording is only required, applying this wavelength is worth considering when something better and life-saving effort can be targeted/achieved. This wavelength demonstrated clear detection of breast tumor progression in animal models *in vivo*. However, applying the same concept in the clinical scenario of breast tumor detection may be challenging unless tested but with appropriate modifications in the device, like including a high-gain preamplifier in case of weak signal strengths and other modifications in the recording strategy. This hypothesis is also supported by the study conducted by Feng et al. in 2016, showcasing the effectiveness of photoacoustic spectral analysis (PASA) as an innovative approach for quantitatively characterizing biological tissues. They have suggested a miniaturized PA probe and its incorporation into the body as minimally invasive for clinical application.^28^

Further, in animal studies, the skin thickness of female mice is 520 ± 30 μm, which consists of epidermis, dermis, and hypodermis.^45^ The thickness of the stratum corneum (outermost layer of skin containing keratin and lipids) is 1–30 μm.^46^ Various proteins in this region absorb at 281 nm and cause pressure waves in the acoustic region. The acoustic waves are mechanical waves that travel deeper into the tissue where the tumor implant is located and reflect back to the PZT detector in physical contact with the skin surface over the tumor. The detected photoacoustic signals contain information about the tumor, which can be analyzed further to assess the tumor progression.

Since the photoacoustic signals were recorded *in vivo*, the spectra were initially preprocessed to remove the unwanted artifacts raised due to the animal vibrations caused by the heartbeat, breathing, etc.^47^ To avoid this artifact, each PA spectrum was recorded with an average of 50 shots of laser pulses (10 Hz × 5 = 5 s).^16^ Similarly, the background spectra were also collected to reduce any signal noise interfering during the experimentation maximally.^16,17,48^ Finally, the concurrent photoacoustic data analysis enabled by machine learning was implemented to demonstrate the potential of photoacoustic spectral analysis in assessing breast tumor progression.

Under machine learning, the preprocessed PA spectra were initially subjected to CWT, a highly redundant transform, to identify their hidden features. The application of CWT in the current study compared to discrete wavelet transform (DWT) was based on its freedom to select wavelets of different scales compared to the limited option by DWT. Further, the computational resources required to compute the CWT and store the coefficients are much larger than the DWT due to the redundancy of CWT. However, the redundancy makes drawing conclusions from the data simpler and interpreting the results better than DWT. Thus, when data analysis aims to discover patterns/hidden information instead of data compression, as in the case of DWT, using redundancy with CWT is more effective. Concomitantly, the selection of the mother wavelet also plays an important role in the accuracy of the machine learning models.^49^ In our study, we have selected “bior 2.6” mother wavelet based on the preliminary analysis of the performance of different mother wavelets and its impact on machine learning models as shown in the Supporting Information and Figure S8.

Further, to select the relevant features from the data for improved machine learning efficiency and to increase the generalization capability of the model, the mRMR algorithm was used. The mRMR algorithm uses mutual information from the features to select only the relevant feature for classification.^50,51^ It was first applied in microarray data to select a small subset of relevant genes out of thousands of genes.^52^ Regarding PAS data belonging to breast and oral cancer samples, the mRMR application demonstrated optimal outcomes through simplified feature selection steps for building input feature matrix.^17,48,53,54^ The mRMR reduces the feature matrix dimension by feature ranking to exclude the redundant features, unlike dimension reduction through transformation to principal components by principal component analysis.^55^ The mRMR algorithm is a powerful feature selection method that can improve the machine learning model’s efficiency, interpretability, and generalization performance. Its ability to identify the most informative and nonredundant features from a large set of features makes it a popular choice in various applications.^56^ In the study presented here, incremental feature selection was performed to select the optimum number of features. The top 20 features were observed to perform best using the SVM model, as shown in Supporting Information Figure S9.

The feature analysis of the data demonstrated that the top 20 spectral features selected using the mRMR algorithm had shown significant differences for different time points of tumor progression under study with significant differences with *p* < 0.001 upon one-way ANOVA. Interestingly, the top features 2, 7, 9, 11, 13, 18, and 20 showed a linear increase/decrease in amplitude of the PA spectra of day 0, 5, 10, 15, and 20 which might be due to the biochemical changes in the tumor tissues as they grow. In an *ex vivo* study conducted by Rodrigues et al., 2021, with a similar set of time points (day 0, 5, 10, 15, and 20 post-tumor induction), they evaluated the corresponding histological patterns demonstrating tumor progression and showed cell death during the establishment of the tumor in the animal (i.e., day 5, 10), a well-established tumor tissue on day 15 and necrotic tissue in day 20, and these changes in the tumor tissues were captured by the PAS *ex vivo* and the metabolomic evidence reported therein suggests variations in tryptophan metabolism during tumor progression concord with the photoacoustic spectroscopic data.^17^ Considering the histological and metabolomic data from the earlier study, the current *in vivo* study demonstrated similar histological findings for day 20 patterns, pleomorphic and abnormal nuclei, and the aggressiveness of the tumor phenotype featured by karyorrhexis, a representative of necrosis corresponding to high-grade tumors.^57^ Therefore, these biological changes that might have occurred during the tumor progression might have contributed to the changes in the PAS features selected, as shown in Figure 5.

After the feature selection, an input feature matrix was built and subjected to SVM-based analysis for data classification. SVM is a powerful machine learning algorithm with several advantages, including its ability to handle highly dimensional data and achieve high accuracy in classification tasks. When applied to photoacoustic spectral data analysis, SVM analysis demonstrated promising outcomes in various application areas such as pathological classification of tissues, biomolecule quantification, etc.^58^ In addition, SVM has a robust kernel function to map input data into a higher-dimensional space with a linear decision boundary even if the data are not linearly separable in the original feature space. The choice of kernel function depends on the data’s nature and the classification task’s complexity. With the right kernel function combination, the SVM is crucial in finding decision boundaries for achieving high classification accuracy. The lower likelihood of overfitting is another advantage of SVM compared to other machine learning algorithms, such as neural networks. This is because SVM aims to maximize the margin between the classes, which reduces the risk of overfitting by keeping the decision boundary as far away from the data points as possible. This makes SVM suitable for analyzing complex data that cannot be separated by simple linear models.^59,60^ As mentioned in the Materials and Methods section, in the current study, multiclass SVM was applied to classify the PA spectra belonging to different time points of tumor progression. There has almost been a consensus that SVM outperforms other classification methods for breast cancer detection. However, an SVM classifier’s performance greatly depends on the proper choice of a kernel function, among other factors.^61,62^

In the current study, we have employed three kernel functions (linear, polynomial, and radial basis functions) and adopted the RBF kernel, which gave a maximum accuracy of 94.5% compared to the linear and polynomial kernels with accuracies of 86% and 91% (Supporting Information Section 3 and Figure S10). This suggested that SVM-RBF is suitable for classification in the current study.

In the current *in vivo* study, SVM analysis was used with a 60–40 scheme for training and testing of the model, which demonstrated improved performance with an overall accuracy of 94.5%, specificity of 100%, and sensitivity of 95, 100, 92.5, and 85% for days 0, 5, 10, 15, and 20, respectively. Further, *k*-fold cross-validation with a 90–10 scheme demonstrated a mean accuracy of 97.5 ± 1.75, ranging from 96 to 100%. This clearly demonstrated that the model is not overfitting. As mentioned in the Materials and Methods section, CWT was implemented for spectral decomposition and analysis to achieve this. CWT uses a single wavelet function with different scales and positions, whereas in the case of an earlier *ex vivo* study conducted by Rodrigues et al., for classifying progressive breast tumor xenograft, wavelet packet transform (WPT) was used utilizing multiple wavelet functions at each level of decomposition to create orthogonal and complete wavelet packets, achieving a promising outcome with SVM-linear multiclass analysis with specificity and sensitivity of 100% for days 5, 10, and 15, and 98% for day 20, respectively, with an overall accuracy of 99%.^17^

In the current study, WPT did not give a similar outcome, possibly due to the interference from animal vibrations under live conditions during PA recordings. In WPT, different mother wavelets were used during transformation analysis, followed by machine learning by SVM. The model’s overall accuracy was 80% despite the least RMS error after reconstruction. Hence, CWT has been used instead of WPT for spectral decomposition and analysis in the current study.

As the mice grow, their skin collagen levels also alter, and spectroscopy-based techniques, including PAS, are suitable to assess such changes.^63,64^ When this alteration is evaluated *in vivo*, it can provide an actual level of collagen on the skin. Thus, when the subcutaneous tumor is grown, the corresponding PAS patterns represent the combined collagen levels of the tumor and the skin. Therefore, in the current study, PAS patterns from control (with only skin) and tumor-bearing animals (skin + tumor) were recorded *in vivo* using a machine learning model to differentiate tumor-specific change and the progression of the tumor. The results showed 100% accuracy of the models, suggesting that the recorded PA signals are tumor-specific.

PA spectral data analysis-based classification of various pathological conditions has gained significant momentum in recent years^27^ because of its sensitivity in capturing minor biochemical changes upon disease progression, unique to different biomolecules. This can further aid in the early diagnosis of diseases in asymptomatic cases^65^ monitoring the response to treatments.^66,67^ Although the spectral data variation in progressive tumor conditions detected by the PAS technique is unique, these are not always identifiable. These necessitate the application of machine learning models to identify the spectral data for its improved efficacy, interpretability, and generalization ability.^68^ Machine learning algorithms such as SVM and mRMR can aid in classifying PA spectra accurately and efficiently, enabling a reliable diagnosis of diseases and therapy responses.

### Significance of the Study in the Clinical Scenario

In an aggressive tumor, necrotic cell death is a common feature when the tumor grows and outgrows blood supply.^69^ A clinical trial conducted by Eugenio et al. in 2009 reported that early invasive breast cancers show a pattern of central necrosis and fibrosis (CNF). The study concluded that the presence of CNF should be routinely reported, as it is an important prognostic and predictive factor in early breast cancer in clinical scenarios.^70^ Also, although current PAS methods have limitations and more research is needed, they may have the potential to improve cancer outcomes by early detection of circulating proteins related to cancer in blood or circulating tumor cells and potentially treating them.^35^

## Conclusions

The current study involved the establishment of a breast tumor xenograft in nude mice via the MCF-7 cell line injection. The tumor progression was evaluated *in vivo* by PAS in machine learning integration. Tumor volume kinetics were used to validate the advancement of breast tumors in nude mice. An additional highlight of the study was the development of an aseptic chamber with PA probe integration to transport tumor-bearing nude mice from the animal house to the laboratory and conduct the PAS experiments *in vivo* at 281 nm excitation using the same chamber in a laboratory setting under germ-free condition. The PA spectra recorded at different time points clearly demonstrated the tumor progression. The multiclass SVM with RBF kernel demonstrated classification performance with the specificity of 100% for day 0 and sensitivity of 95, 100, 92.5, and 85% for days 0, 5, 10, 15, and 20, respectively, with an overall accuracy of 94.5%. The photoacoustic signal analysis-based classification of breast tumor progression has the potential to provide a noninvasive and label-free diagnosis of the disease, as demonstrated in the current study, as proof of principle. In addition, PA signal analysis is simple and robust in application to preclinical and clinical settings. To the best of our knowledge, this is the first study to report the detection of breast tumor progression *in vivo* at different time points in the same animals and to classify tumor progression based on photoacoustic spectral analysis. The study provides information on biochemical/molecular-based functional imaging of breast tumor progression reflected in photoacoustic signals under study. As PAS captures biochemical changes, information on early disease onset through biomolecular mapping can be obtained.

## Supporting Information Available

Data relating to optimizing the probe, flowchart of preprocessing steps, typical PAS patterns of each preprocessing steps belonging to all time point groups, selection of mother wavelet, incremental feature selection, and cross-validation (Figures S1–10) (PDF)

## References

[ref1] Sung H.; Ferlay J.; Siegel R. L.; Laversanne M.; Soerjomataram I.; Jemal A.; Bray F. Global Cancer Statistics 2020: GLOBOCAN Estimates of Incidence and Mortality Worldwide for 36 Cancers in 185 Countries. Ca-Cancer J. Clin. 2021, 71 (3), 209–249. 10.3322/caac.21660. 33538338

[ref2] Migowski A. A Detecção Precoce Do Câncer de Mama e a Interpretação Dos Resultados de Estudos de Sobrevida. Ciênc. Saúde Coletiva 2015, 20 (4), 1309 10.1590/1413-81232015204.17772014. 25923642

[ref3] Zhang R.; Zhao L.; Zhao C.; Wang M.; Liu S.; Li J.; Zhao R.; Wang R.; Yang F.; Zhu L.; He X.; Li C.; Jiang Y.; Yang M. Exploring the Diagnostic Value of Photoacoustic Imaging for Breast Cancer: The Identification of Regional Photoacoustic Signal Differences of Breast Tumors. Biomed. Opt. Express 2021, 12 (3), 1407 10.1364/BOE.417056. 33796362 PMC7984795

[ref4] Wang L. Early Diagnosis of Breast Cancer. Sensors 2017, 17 (7), 1572 10.3390/s17071572. 28678153 PMC5539491

[ref5] Ahn H.; Song H.; Shin D.-M.; Kim K.; Choi J. Emerging Optical Spectroscopy Techniques for Biomedical Applications—A Brief Review of Recent Progress. Appl. Spectrosc. Rev. 2018, 53 (2–4), 264–278. 10.1080/05704928.2017.1324877.

[ref6] Yang M.; Mahanty A.; Jin C.; Wong A. N. N.; Yoo J. S. Label-Free Metabolic Imaging for Sensitive and Robust Monitoring of Anti-CD47 Immunotherapy Response in Triple-Negative Breast Cancer. J. Immunother. Cancer 2022, 10 (9), e005199 10.1136/jitc-2022-005199. 36096527 PMC9472253

[ref7] Matsui T.; Iwasa A.; Mimura M.; Taniguchi S.; Sudo T.; Uchida Y.; Kikuta J.; Morizono H.; Horii R.; Motoyama Y.; Morii E.; Ohno S.; Kiyota Y.; Ishii M. Label-free Multiphoton Excitation Imaging as a Promising Diagnostic Tool for Breast Cancer. Cancer Sci. 2022, 113 (8), 2916–2925. 10.1111/cas.15428. 35579268 PMC9357641

[ref8] Duan G.; Zhang J.; Wei Z.; Wang X.; Zeng J.; Wu S.; Hu C.; Wen L. Intraoperative Diagnosis of Early Lymphatic Metastasis Using Neodymium-Based Rare-Earth NIR-II Fluorescence Nanoprobe. Nanoscale Adv. 2023, 5 (16), 4240–4249. 10.1039/D3NA00254C. 37560436 PMC10408585

[ref9] Zhu Y.-Y.; Song L.; Zhang Y.-Q.; Liu W.-L.; Chen W.-L.; Gao W.-L.; Zhang L.-X.; Wang J.-Z.; Ming Z.-H.; Zhang Y.; Zhang G.-J. Development of a Rare Earth Nanoprobe Enables in Vivo Real-Time Detection of Sentinel Lymph Node Metastasis of Breast Cancer Using NIR-IIb Imaging. Cancer Res. 2023, 83, 3428 10.1158/0008-5472.CAN-22-3432. 37540231 PMC10570679

[ref10] Park B.; Oh D.; Kim J.; Kim C. Functional Photoacoustic Imaging: From Nano- and Micro- to Macro-Scale. Nano Converg 2023, 10 (1), 29 10.1186/s40580-023-00377-3. 37335405 PMC10279631

[ref11] Lengenfelder B.; Mehari F.; Hohmann M.; Heinlein M.; Chelales E.; Waldner M. J.; Klämpfl F.; Zalevsky Z.; Schmidt M. Remote Photoacoustic Sensing Using Speckle-Analysis. Sci. Rep 2019, 9 (1), 1057 10.1038/s41598-018-38446-x. 30705342 PMC6355860

[ref12] Wang L. V.; Hu S. Photoacoustic Tomography: In Vivo Imaging from Organelles to Organs. Science 2012, 335 (6075), 1458–1462. 10.1126/science.1216210. 22442475 PMC3322413

[ref13] Wang L. V. Multiscale Photoacoustic Microscopy and Computed Tomography. Nat. Photonics 2009, 3 (9), 503–509. 10.1038/nphoton.2009.157. 20161535 PMC2802217

[ref14] Tsang V. T. C.; Li X.; Wong T. T. W. A Review of Endogenous and Exogenous Contrast Agents Used in Photoacoustic Tomography with Different Sensing Configurations. Sensors 2020, 20 (19), 5595 10.3390/s20195595. 33003566 PMC7582683

[ref15] Upputuri P. K.; Pramanik M. Performance Characterization of Low-Cost, High-Speed, Portable Pulsed Laser Diode Photoacoustic Tomography (PLD-PAT) System. Biomed. Opt. Express 2015, 6 (10), 4118 10.1364/BOE.6.004118. 26504659 PMC4605068

[ref16] Priya M.; Satish Rao B. S.; Chandra S.; Datta A.; Nayak S. G.; Mahato K. K. Monitoring Breast Tumor Progression by Photoacoustic Measurements: A Xenograft Mice Model Study. J. Biomed. Opt. 2015, 20 (10), 105002 10.1117/1.JBO.20.10.105002. 26442962

[ref17] Rodrigues J.; Amin A.; Raghushaker C. R.; Chandra S.; Joshi M. B.; Prasad K.; Rai S.; Nayak S. G.; Ray S.; Mahato K. K. Exploring Photoacoustic Spectroscopy-Based Machine Learning Together with Metabolomics to Assess Breast Tumor Progression in a Xenograft Model Ex Vivo. Lab. Invest. 2021, 101 (7), 952–965. 10.1038/s41374-021-00597-3. 33875792 PMC8214996

[ref18] Liu C.; Wang L. Functional Photoacoustic Microscopy of Hemodynamics: A Review. Biomed. Eng. Lett. 2022, 12 (2), 97–124. 10.1007/s13534-022-00220-4. 35529339 PMC9046529

[ref19] Du J.; Yang S.; Qiao Y.; Lu H.; Dong H. Recent Progress in Near-Infrared Photoacoustic Imaging. Biosens. Bioelectron. 2021, 191, 113478 10.1016/j.bios.2021.113478. 34246125

[ref20] Li J.; Chen Y.; Ye W.; Zhang M.; Zhu J.; Zhi W.; Cheng Q. Molecular Breast Cancer Subtype Identification Using Photoacoustic Spectral Analysis and Machine Learning at the Biomacromolecular Level. Photoacoustics 2023, 30, 100483 10.1016/j.pacs.2023.100483. 37063308 PMC10090435

[ref21] Jo J.; Folz J.; Gonzalez M. E.; Paolì A.; Eido A.; Salfi E.; Tekula S.; Andò S.; Caruso R.; Kleer C. G.; Wang X.; Kopelman R. Personalized Oncology by In Vivo Chemical Imaging: Photoacoustic Mapping of Tumor Oxygen Predicts Radiotherapy Efficacy. ACS Nano 2023, 17, 4396 10.1021/acsnano.2c09502. 36847392 PMC10149113

[ref22] Lin L.; Hu P.; Shi J.; Appleton C. M.; Maslov K.; Li L.; Zhang R.; Wang L. V. Single-Breath-Hold Photoacoustic Computed Tomography of the Breast. Nat. Commun. 2018, 9 (1), 2352 10.1038/s41467-018-04576-z. 29907740 PMC6003984

[ref23] Allen T. J.; Hall A.; Dhillon A. P.; Owen J. S.; Beard P. C. Spectroscopic Photoacoustic Imaging of Lipid-Rich Plaques in the Human Aorta in the 740 to 1400 Nm Wavelength Range. J. Biomed. Opt. 2012, 17 (6), 061209 10.1117/1.JBO.17.6.061209. 22734739

[ref24] Manwar R.; McGuire L.; Shoo A.; Charbel F.; Pillers D.-A.; Avanaki K. Cerebral Blood Oxygenation Measurement in Sheep Brain In-Vivo Using Transfontanelle Photoacoustic Spectroscopy. In Photons Plus Ultrasound: Imaging and Sensing 2022; Oraevsky A. A.; Wang L. V., Eds.; SPIE, 2022; p 163.

[ref25] Burgholzer P.; Bauer-Marschallinger J.; Reitinger B.; Berer T. Resolution Limits in Photoacoustic Imaging Caused by Acoustic Attenuation. J. Imaging 2019, 5 (1), 13 10.3390/jimaging5010013. 34465707 PMC8320872

[ref26] Yao J.; Wang L. V. Recent Progress in Photoacoustic Molecular Imaging. Curr. Opin. Chem. Biol. 2018, 45, 104–112. 10.1016/j.cbpa.2018.03.016. 29631120 PMC6076847

[ref27] Steinberg I.; Huland D. M.; Vermesh O.; Frostig H. E.; Tummers W. S.; Gambhir S. S. Photoacoustic Clinical Imaging. Photoacoustics 2019, 14, 77–98. 10.1016/j.pacs.2019.05.001. 31293884 PMC6595011

[ref28] Feng T.; Li Q.; Zhang C.; Xu G.; Guo L. J.; Yuan J.; Wang X. Characterizing Cellular Morphology by Photoacoustic Spectrum Analysis with an Ultra-Broadband Optical Ultrasonic Detector. Opt. Express 2016, 24 (17), 19853 10.1364/OE.24.019853. 27557261 PMC5025227

[ref29] Deng H.; Qiao H.; Dai Q.; Ma C. Deep Learning in Photoacoustic Imaging: A Review. J. Biomed. Opt. 2021, 26 (04), 040901 10.1117/1.JBO.26.4.040901. 33837678 PMC8033250

[ref30] Feldman A. T.; Wolfe D. Tissue Processing and Hematoxylin and Eosin Staining. Methods Mol. Biol. 2014, 1180, 31–43. 10.1007/978-1-4939-1050-2_3. 25015141

[ref31] Too J.; Abdullah A. R.; Saad N. M.; Ali N. M.; Musa H. A Detail Study of Wavelet Families for EMG Pattern Recognition. Int. J. Electr. Comput. Eng. 2018, 8 (6), 4221 10.11591/ijece.v8i6.pp4221-4229.

[ref32] Ding C.; Peng H. Minimum Redundancy Feature Selection from Microarray Gene Expression Data. In Computational Systems Bioinformatics; CSB2003. Proceedings of the 2003 IEEE Bioinformatics Conference. CSB2003; IEEE Comput. Soc, 2003; Vol. 3, pp 523–528. 10.1142/s0219720005001004 15852500

[ref33] Pisner D. A.; Schnyer D. M. Support Vector Machine. Mach. Learn. 2020, 101–121. 10.1016/B978-0-12-815739-8.00006-7.

[ref34] Wilkinson L.; Gathani T. Understanding Breast Cancer as a Global Health Concern. Br J. Radiol 2022, 95 (1130), 20211033 10.1259/bjr.20211033. 34905391 PMC8822551

[ref35] Veverka M.; Menozzi L.; Yao J. The Sound of Blood: Photoacoustic Imaging in Blood Analysis. Med. Nov. Technol. Devices 2023, 18, 100219 10.1016/j.medntd.2023.100219. 37538444 PMC10399298

[ref36] Galanzha E.; Zharov V. Circulating Tumor Cell Detection and Capture by Photoacoustic Flow Cytometry in Vivo and Ex Vivo. Cancers 2013, 5 (4), 1691–1738. 10.3390/cancers5041691. 24335964 PMC3875961

[ref37] Mahato K. K.; Rodrigues J.; Ray S.; Chandra S. PHotoacoustic Probe for Detecting Cancer Or Other Pathological Disorder in Breast. Indian Patent Application Number: IN202341018911, March 2023.

[ref38] Laufer J.; Jathoul A.; Pule M.; Beard P. In Vitro Characterization of Genetically Expressed Absorbing Proteins Using Photoacoustic Spectroscopy. Biomed. Opt. Express 2013, 4 (11), 2477 10.1364/BOE.4.002477. 24298408 PMC3829541

[ref39] He Q.; Zhang Q.; Cao W.; Yin T.; Zhao S.; Yin X.; Zhao H.; Tao W. Detecting Trace of Mercury Ions in Water Using Photoacoustic Method Enhanced by Gold Nanospheres. Microchem. J. 2019, 150, 104058 10.1016/j.microc.2019.104058.

[ref40] Waasem N.; Fieberg S.; Hauser J.; Gomes G.; Haertle D.; Kühnemann F.; Buse K. Photoacoustic Absorption Spectrometer for Highly Transparent Dielectrics with Parts-per-Million Sensitivity. Rev. Sci. Instrum. 2013, 84 (2), 023109 10.1063/1.4792724. 23464197

[ref41] Cheng A.; Kim Y.; Itsarachaiyot Y.; Zhang H. K.; Weiss C. R.; Taylor R. H.; Boctor E. M. Photoacoustic-Based Catheter Tracking: Simulation, Phantom, and in Vivo Studies. J. Med. Imaging 2018, 5 (02), 021223 10.1117/1.JMI.5.2.021223. PMC5871614 29594184

[ref42] Dangi A.; Cheng C. Y.; Agrawal S.; Tiwari S.; Datta G. R.; Benoit R. R.; Pratap R.; Trolier-Mckinstry S.; Kothapalli S.-R. A Photoacoustic Imaging Device Using Piezoelectric Micromachined Ultrasound Transducers (PMUTs). IEEE Trans. Ultrason., Ferroelect., Freq. Control 2020, 67 (4), 801–809. 10.1109/TUFFC.2019.2956463. PMC7224331 31794394

[ref43] Wang H.; Chen Z.; Yang H.; Jiang H.; Xie H. A Ceramic PZT-Based PMUT Array for Endoscopic Photoacoustic Imaging. J. Microelectromech. Syst. 2020, 29 (5), 1038–1043. 10.1109/JMEMS.2020.3010773. 33746476 PMC7978059

[ref44] Wang H.; Yang H.; Chen Z.; Zheng Q.; Jiang H.; Feng P. X.-L.; Xie H. Development of Dual-Frequency PMUT Arrays Based on Thin Ceramic PZT for Endoscopic Photoacoustic Imaging. J. Microelectromech. Syst. 2021, 30 (5), 770–782. 10.1109/JMEMS.2021.3096733. 35528228 PMC9075345

[ref45] Sabino C. P.; Deana A. M.; Yoshimura T. M.; da Silva D. F. T.; França C. M.; Hamblin M. R.; Ribeiro M. S. The Optical Properties of Mouse Skin in the Visible and near Infrared Spectral Regions. J. Photochem. Photobiol. B 2016, 160, 72–78. 10.1016/j.jphotobiol.2016.03.047. 27101274 PMC4899976

[ref46] Ge N.; Zhang Y.; Zhang H.; Cheng L.; Shi X. Extraction and Characterization of Keratin and Keratin Hydrogels from Wasted Rabbit Hair. J. Phys.: Conf. Ser. 2021, 1790 (1), 012008 10.1088/1742-6596/1790/1/012008.

[ref47] Jia H.; Liu J.; Fang T.; Zhou Z.; Li R.; Yin W.; Qian Y.; Wang Q.; Zhou W.; Liu C.; Sun D.; Chen X.; Ouyang Z.; Dong J.; Wang Y.; Yue S. The Role of Altered Lipid Composition and Distribution in Liver Fibrosis Revealed by Multimodal Nonlinear Optical Microscopy. Sci. Adv. 2023, 9 (2), eabq2937 10.1126/sciadv.abq2937. 36638165 PMC9839333

[ref48] Raghushaker C. R.; Rodrigues J.; Nayak S. G.; Ray S.; Urala A. S.; Satyamoorthy K.; Mahato K. K. Fluorescence and Photoacoustic Spectroscopy-Based Assessment of Mitochondrial Dysfunction in Oral Cancer Together with Machine Learning: A Pilot Study. Anal. Chem. 2021, 93 (49), 16520–16527. 10.1021/acs.analchem.1c03650. 34846862

[ref49] Pothisarn C.; Klomjit J.; Ngaopitakkul A.; Jettanasen C.; Asfani D. A.; Negara I. M. Y. Comparison of Various Mother Wavelets for Fault Classification in Electrical Systems. Appl. Sci. 2020, 10 (4), 1203 10.3390/app10041203.

[ref50] Verleysen M.; Rossi F.; François D. Advances in Feature Selection with Mutual Information. 2009, pp 52–69 10.1007/978-3-642-01805-3_4.

[ref51] Vergara J. R.; Estévez P. A. A Review of Feature Selection Methods Based on Mutual Information. Neural Comput. Appl. 2014, 24 (1), 175–186. 10.1007/s00521-013-1368-0.

[ref52] Ding C.; Peng H. Minimum Redundancy Feature Selection from Microarray Gene Expression Data. J. Bioinf. Comput. Biol. 2005, 03 (02), 185–205. 10.1142/S0219720005001004. 15852500

[ref53] Rodrigues J.; Akhil K. A.; Mahato K. K. Discriminatory Potential of Photoacoustic Spectroscopic Fingerprints Integrated with Machine Learning to Distinguish between Different Organs: Ex Vivo. In Frontiers in Optics + Laser Science 2022 (FIO, LS); Optica Publishing Group: Washington, D.C., 2022; p FTh3B.5.

[ref54] Rodrigues J.; Amin A.; Chandra S.; Nayak G. S.; Ray S.; Satyamoorthy K.; Mahato K. K. Detecting Breast Tumor by Photoacoustic Spectroscopy Integrated Machine Learning: A Comparison of Statistical and Algorithm Based Features. In Frontiers in Optics + Laser Science 2021; Optica Publishing Group: Washington, D.C., 2021; p JW7A.10.

[ref55] Saeys Y.; Inza I.; Larrañaga P. A Review of Feature Selection Techniques in Bioinformatics. Bioinformatics 2007, 23 (19), 2507–2517. 10.1093/bioinformatics/btm344. 17720704

[ref56] Ding C.; Peng H. Minimum Redundancy Feature Selection from Microarray Gene Expression Data. In Computational {Systems} {Bioinformatics}. {CSB2003}. {Proceedings} of the 2003 {IEEE} {Bioinformatics} {Conference}. {CSB2003}; IEEE Comput. Soc: Stanford, CA, USA, 2003; pp 523–528. 10.1142/s0219720005001004 15852500

[ref57] Wang S.; Rong R.; Yang D. M.; Fujimoto J.; Yan S.; Cai L.; Yang L.; Luo D.; Behrens C.; Parra E. R.; Yao B.; Xu L.; Wang T.; Zhan X.; Wistuba I. I.; Minna J.; Xie Y.; Xiao G. Computational Staining of Pathology Images to Study the Tumor Microenvironment in Lung Cancer. Cancer Res. 2020, 80 (10), 2056–2066. 10.1158/0008-5472.CAN-19-1629. 31915129 PMC7919065

[ref58] Huang S.; Nianguang C. A. I.; Penzuti Pacheco P.; Narandes S.; Wang Y.; Wayne X. U. Applications of Support Vector Machine (SVM) Learning in Cancer Genomics. Cancer Genomics Proteomics 2018, 15 (1), 41–51. 10.21873/cgp.20063. 29275361 PMC5822181

[ref59] Sabat-Tomala A.; Raczko E.; Zagajewski B. Comparison of Support Vector Machine and Random Forest Algorithms for Invasive and Expansive Species Classification Using Airborne Hyperspectral Data. Remote Sens. 2020, 12 (3), 516 10.3390/rs12030516.

[ref60] Huang S.; Cai N.; Pacheco P. P.; et al. Applications of Support Vector Machine (SVM) Learning in Cancer Genomics. Cancer Genomics Proteomics 2018, 15 (1), 41–51. 10.21873/cgp.20063. 29275361 PMC5822181

[ref61] Hussain M.; Wajid S. K.; Elzaart A.; Berbar M. In A Comparison of SVM Kernel Functions for Breast Cancer Detection, 2011 Eighth International Conference Computer Graphics, Imaging and Visualization, IEEE, 2011; pp 145–150.

[ref62] Karal O. In Performance Comparison of Different Kernel Functions in SVM for Different k Value in K-Fold Cross-Validation, 2020 Innovations in Intelligent Systems and Applications Conference (ASYU); IEEE, 2020; pp 1–5.

[ref63] Boyer B.; Kern P.; Fourtanier A.; Labat-Robert J. Age-Dependent Variations of the Biosyntheses of Fibronectin and Fibrous Collagens in Mouse Skin. Exp. Gerontol. 1991, 26 (4), 375–383. 10.1016/0531-5565(91)90049-R. 1936196

[ref64] Robert L.; Labat-Robert J. Morphogenesis, Aging, and Repair of the Connective Tissues. Facial Plast. Surg. 1989, 6 (01), 1–7. 10.1055/s-2008-1064703.

[ref65] Nyayapathi N.; Xia J. Photoacoustic Imaging of Breast Cancer: A Mini Review of System Design and Image Features. J. Biomed. Opt. 2019, 24 (12), 1 10.1117/1.JBO.24.12.121911. PMC7005545 31677256

[ref66] Mehrmohammadi M.; Joon Yoon S.; Yeager D.; et al. Photoacoustic Imaging for Cancer Detection and Staging. Curr. Mol. Imaging 2013, 2 (1), 89–105. 10.2174/2211555211302010010. 24032095 PMC3769095

[ref67] Lefebvre T. L.; Brown E.; Hacker L.; Else T.; Oraiopoulou M.-E.; Tomaszewski M. R.; Jena R.; Bohndiek S. E. The Potential of Photoacoustic Imaging in Radiation Oncology. Front. Oncol. 2022, 12, 803777 10.3389/fonc.2022.803777. 35311156 PMC8928467

[ref68] R Gharieb R. Photoacoustic Imaging for Cancer Diagnosis: A Breast Tumor Example. In Photoacoustic Imaging - Principles, Advances and Applications; IntechOpen, 2020.

[ref69] Yamamoto A.; Huang Y.; Krajina B. A.; McBirney M.; Doak A. E.; Qu S.; Wang C. L.; Haffner M. C.; Cheung K. J. Metastasis from the Tumor Interior and Necrotic Core Formation Are Regulated by Breast Cancer-Derived Angiopoietin-like 7. Proc. Natl. Acad. Sci. U.S.A. 2023, 120 (10), e2214888120 10.1073/pnas.2214888120. 36853945 PMC10013750

[ref70] Maiorano E.; Regan M. M.; Viale G.; Mastropasqua M. G.; Colleoni M.; Castiglione-Gertsch M.; Price K. N.; Gelber R. D.; Goldhirsch A.; Coates A. S. Prognostic and Predictive Impact of Central Necrosis and Fibrosis in Early Breast Cancer: Results from Two International Breast Cancer Study Group Randomized Trials of Chemoendocrine Adjuvant Therapy. Breast Cancer Res. Treat. 2010, 121 (1), 211–218. 10.1007/s10549-009-0360-y. 19280340 PMC3588888

[ref71] Yang T.; Jin Y.; Neogi A. Acoustic Attenuation and Dispersion in Fatty Tissues and Tissue Phantoms Influencing Ultrasound Biomedical Imaging. ACS Omega 2023, 8 (1), 1319–1330. 10.1021/acsomega.2c06750. 36643513 PMC9835773

