# Supplementary material for: Machine Learning Enabled Photoacoustic Spectroscopy for Noninvasive Assessment of Breast Tumor Progression In Vivo: A Preclinical Study
Source: ACS Sens. 2024 Jan 30;9(2):589–601. doi: 10.1021/acssensors.3c01085 (PMC10897932; doi:10.1021/acssensors.3c01085)
Supplement: Supplementary file 1 — se3c01085_si_001.pdf [file se3c01085_si_001.pdf]

Supplementary Information

Machine learning enabled photoacoustic spectroscopy for noninvasive assessment of breast tumor progression *in vivo:* A preclinical study.

Jackson Rodrigues[1], Ashwini Amin[2], Subhash Chandra[1], Nitufa J Mulla[1], G Subramanya Nayak[3], Sharada Rai[4], Satadru Ray[5], Krishna Kishore Mahato[1]*

1. Department of Biophysics, Manipal School of Life Sciences, Manipal Academy of Higher Education, Karnataka, Manipal, 576 104, India.
2. Department of Computer Science and Engineering, Manipal Institute of Technology, Manipal Academy of Higher Education, Manipal, 576104, India.
3. Department of Electronics and Communication, Manipal Institute of Technology, Manipal Academy of Higher Education, Manipal, 576104, India.
4. Department of Pathology, Kasturba Medical College Mangalore, Manipal Academy of Higher Education, Karnataka, Manipal, 576 104, India.
5. Department of Surgery, Kasturba Medical College, Manipal Academy of Higher Education, Karnataka, Manipal, 576 104, India.

* Corresponding author, email – **kkmahato@gmail.com;** mahato.kk@manipal.edu

1. Optimization of PA probe:

To investigate the backing up of the transducer, during optimization of the in-house developed PA probe, tryptophan solution was used as the standard. Initially, 5 mM tryptophan solution was prepared using deionized water and used for the recording of photoacoustic signals at 281nm pulsed laser excitations using the probe developed. The tryptophan solution was kept in a quartz cuvette and held in contact with the probe surface-PZT-detector assembly for the recording of the photoacoustic signals. The typical photoacoustic spectrum recorded at 281nm pulsed laser excitation of tryptophan using the probe is shown in Figure S1A. Subsequently, the recording was taken with deionized water is shown in Figure S1B. This data suggests that the PA probe assembly and the transducer housing is stable and sturdy, and the PA signals obtained during the 281 nm laser excitation are absolutely from absorption from samples and not from any nonspecific pressure variations.

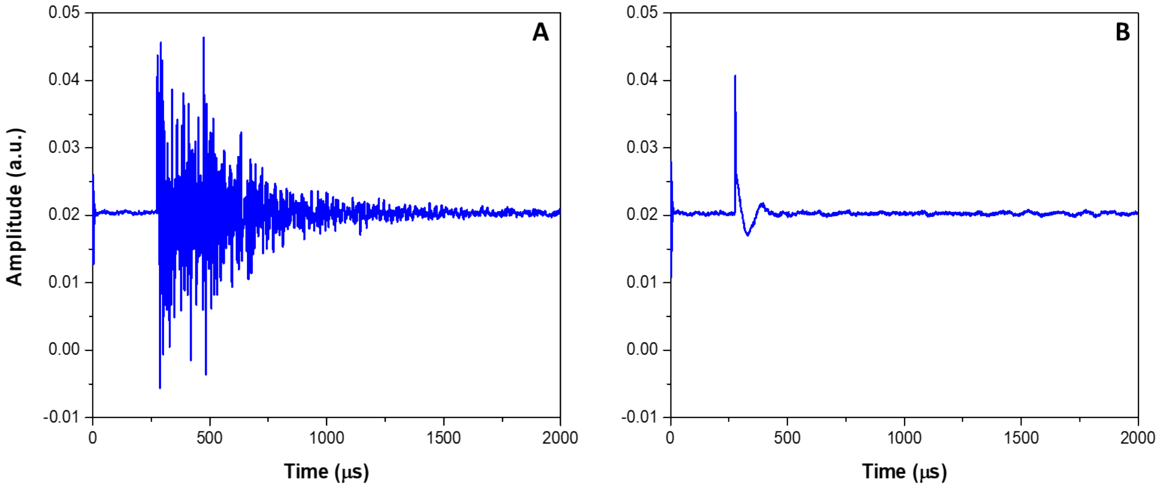

Figure S1: Typical, raw photoacoustic spectra of (A) tryptophan solution in deionized water and (B) the deionized water alone at 281 nm excitations.

2. Data Analysis:

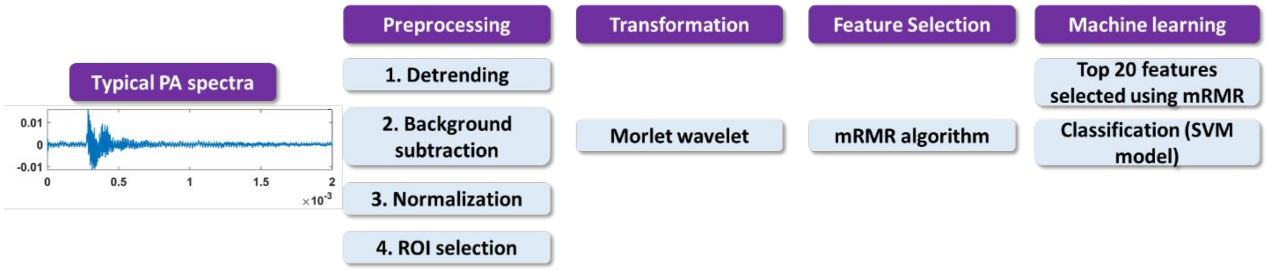

Figure S2: Data analysis workflow used in the study.

Preprocessing of the in vivo PA spectra:

The PA spectra generated by the 10 Hz pulsed laser excitation of 6 ns pulse width was initially averaged to nullify the electrical noise. The averaged signal (5 seconds = 50 laser pulses) was amplified using a preamplifier. The amplified signals were recorded in time-domain as a text file using a digital oscilloscope. The PA spectra were recorded from the tumor regions of tumor bearing animals and flank regions of control animals by exciting them using 281 nm pulsed laser excitation were considered as signals and the spectra that were recorded with no laser excitation (laser blocked condition) were considered as background.

The background and the signals were recorded at 2.5e6 sample rate from 0-2 ms. All the signals along with background, were initially detrended to remove the trends from time series data. The detrended signals were then background subtracted and the subsequent normalization was done. In this study we have normalized each background subtracted spectra with respect to its maximum value. The normalization was done to visualize the PA spectral patterns which should be irrespective of their amplitude of the signals. after the normalization. A common region of interest was selected (0.275-3.2 ms) for each spectrum and were further subjected to wavelet transformation [1–3] (Priya M et al., 2015, Rodrigues et al., 2021, Raghushaker et al., 2022).

Wavelet transformation:

The CWT is obtained using the analytic Morse wavelet with the symmetry parameter (3, time-bandwidth product – 60). In an octave, CWT uses 10 voices, depending on the energy spread of wavelet in time-frequency, the scales (min and max) are decided automatically. L1 normalization is used in cwt which shows more accurate representation of the signal. [the description was obtained from the following link - https://in.mathworks.com/help/wavelet/ref/cwt.html].

The schematic representation of overall study/data analysis strategy is shown in **figure S2**. The preprocessing steps of time-domain PA spectra along with representative spectra from each time point belonging to day 0th, 5th, 10th, 15th, and 20th, is shown in **figures S3 to S7** respectively.

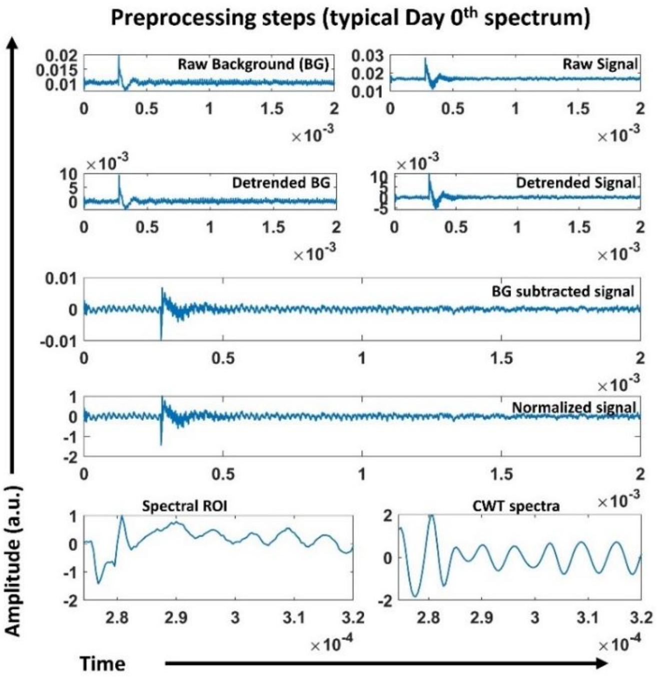

Figure S3: The preprocessing steps of time-domain PA spectra along with a typical PA spectrum from Day 0.

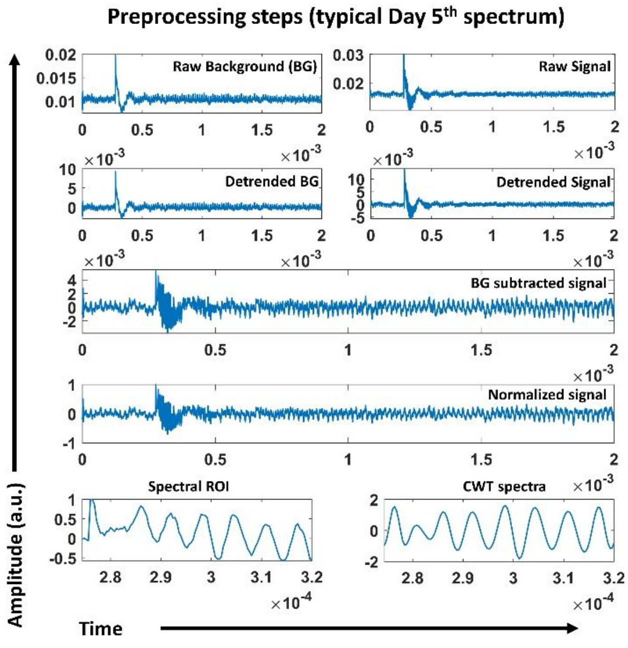

Figure S4: The preprocessing steps of time-domain PA spectra along with a typical PA spectrum from Day 5.

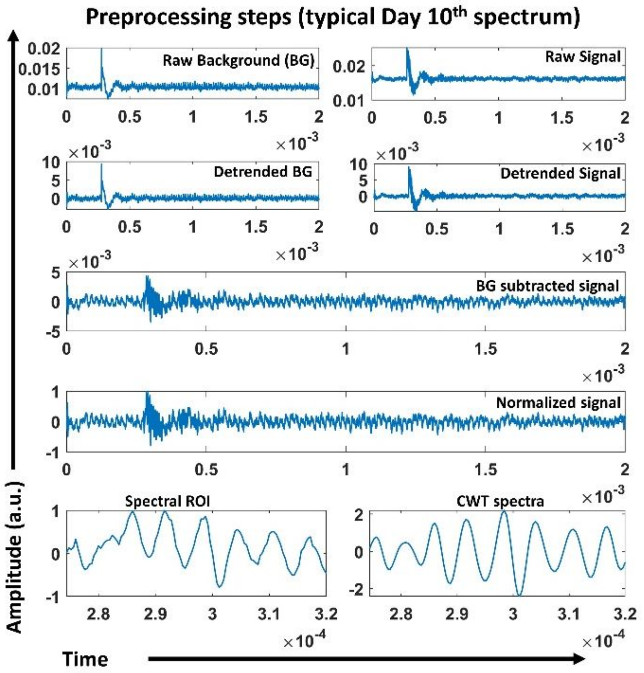

Figure S5: The preprocessing steps of time-domain PA spectra along with a typical PA spectrum from Day 10.

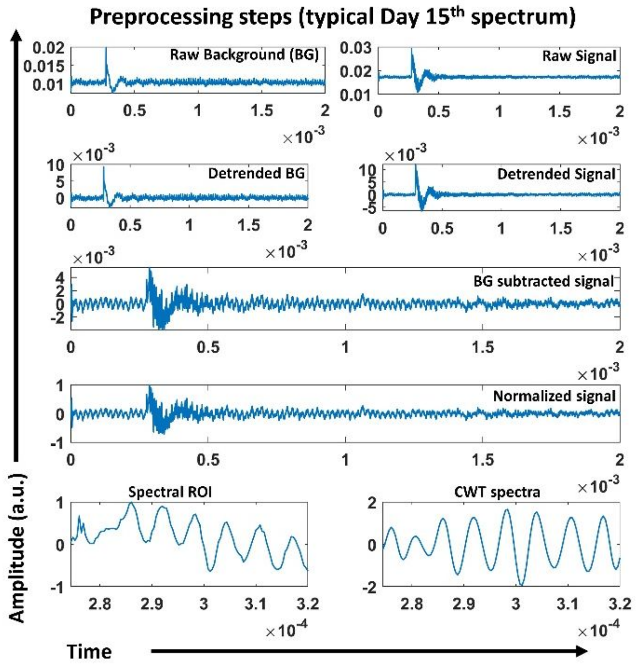

Figure S6: The preprocessing steps of time-domain PA spectra along with a typical PA spectrum from Day 15.

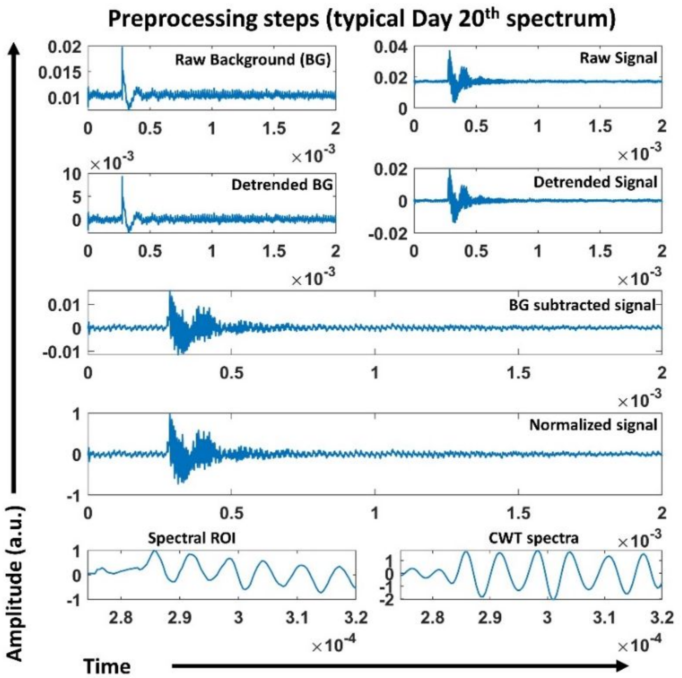

Figure S7: The preprocessing steps of time-domain PA spectra along with a typical PA spectrum from Day 20.

3. Classification

Selection of mother wavelet and machine learning combination

Under the current study, a suitable mother wavelet and a suitable classifier was selected based on their coefficient values as shown in **Figure S8.** The preprocessed PA signals in the current study were subjected to different families of mother wavelets (Daubechies, symlets, coiflets, biorthogonal), for feature selection, followed by classification using different machine learning algorithms. For classification, different classifiers were used for each mother wavelet. It was found that, the mother wavelet 'bior2.6' and the machine learning classifier SVM-RBF showed optimal performance for the classification of the photoacoustic spectral data. As a result, we used this combination to classify the in vivo photoacoustic spectral data to assess the progression of breast tumor in athymic nude mice using MCF-7 cell line derived breast tumor xenograft.

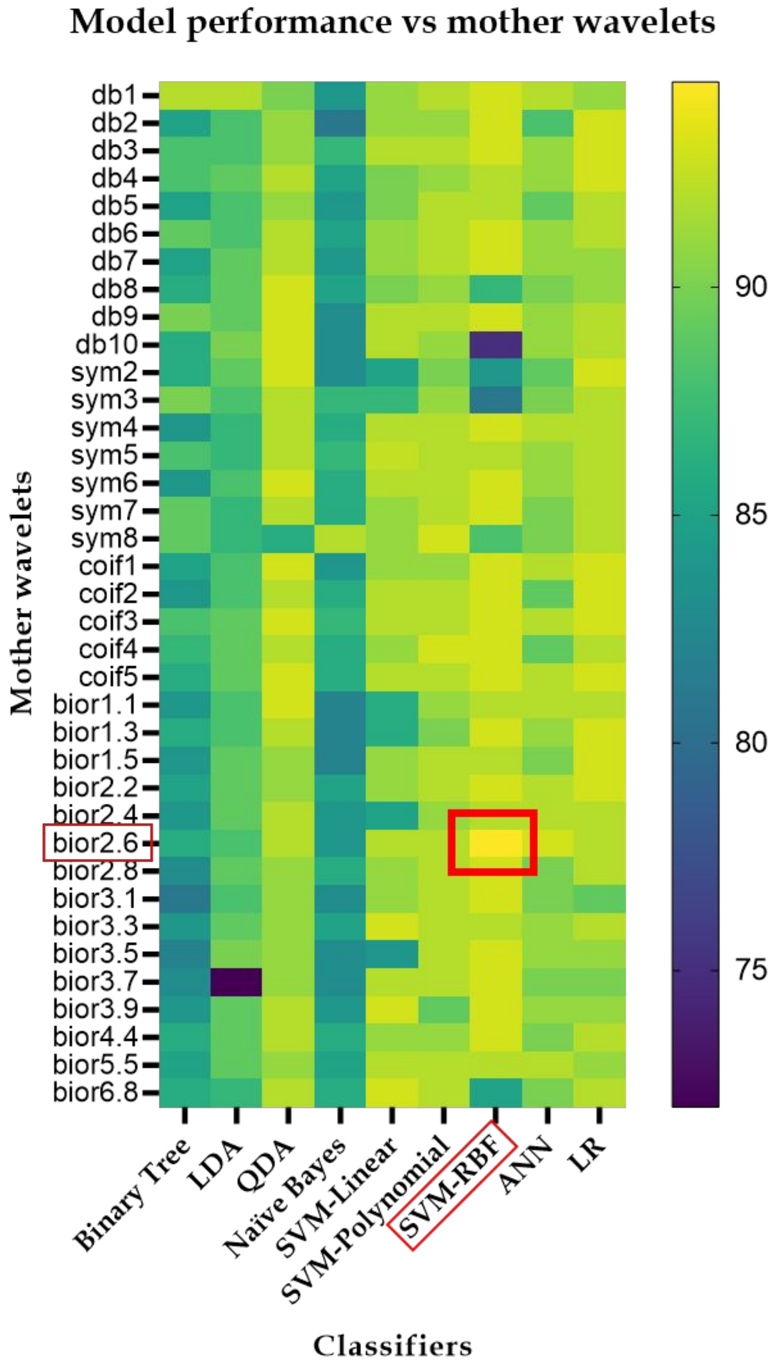

Figure S8: Different mother wavelets (Y-Axis) vs classifiers (X-Axis) with their performance with color bar representing from 72% (least) to 96% (highest). Color strength represents accuracy values starting from 72% (least) to 96% (highest).The best mother wavelet and the corresponding machine learning algorithm has been marked with red box with highest model accuracy of 96%.

Incremental features selection:

To select the optimum number of features that were ranked using the mRMR algorithm, the incremental feature selection was performed starting from the top 1 feature till top 50 features as an input to the SVM model incrementally with 10 iterations each time a feature was added to the model. It was observed that, after 3 features, the accuracy of the model crossed 90% and remained almost same throughout. The task was to select the minimum number of features required for the best performance of the ML model. Interestingly, top 20 features showed relatively best performance compared to any lesser number of features, and more than 20 number of features did not yield much of improvement in the accuracy as shown in **Figure S9**. Therefore, in the current study, the top 20 features were considered as optimum number of features for input feature matrix.

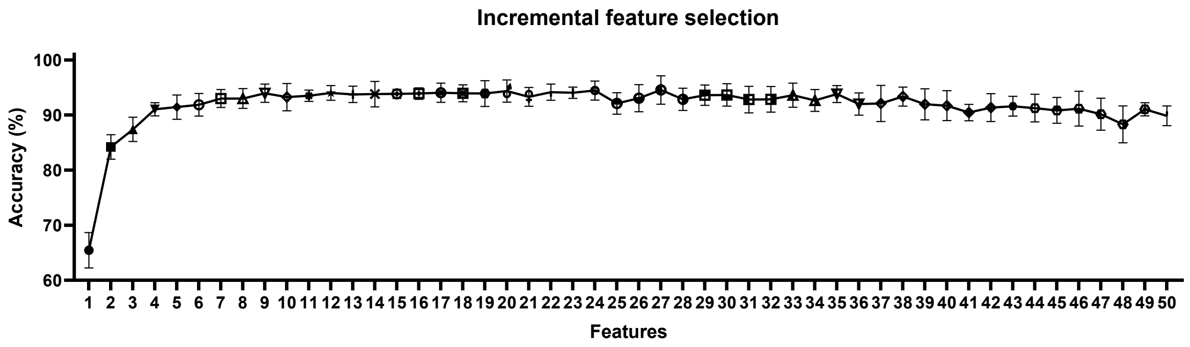

Figure S9*: The varying mean classification accuracy of SVM model with incremental features from top 1 to 50 top ranked features obtained by mRMR algorithm.*

In the current study, we have employed three kernel functions (linear, polynomial, and radial basis function) and adopted the RBF kernel which gave maximum accuracy of 94.5% compared to linear (86%) and polynomial (91%) with 60% training and 40% testing of data sets. For linear kernel model of accuracy 86%, upon k-fold cross validation (k=10) using 90% training vs 10% testing for every cluster, it provided a mean accuracy of 89.8% and for polynomial kernel an accuracy of 96%, whereas RBF kernel showed 97.8 % as shown in **figure S10**. This suggested that the SVM-RBF that was trained was more generalized model compared to the rest (linear and polynomial) of the models.

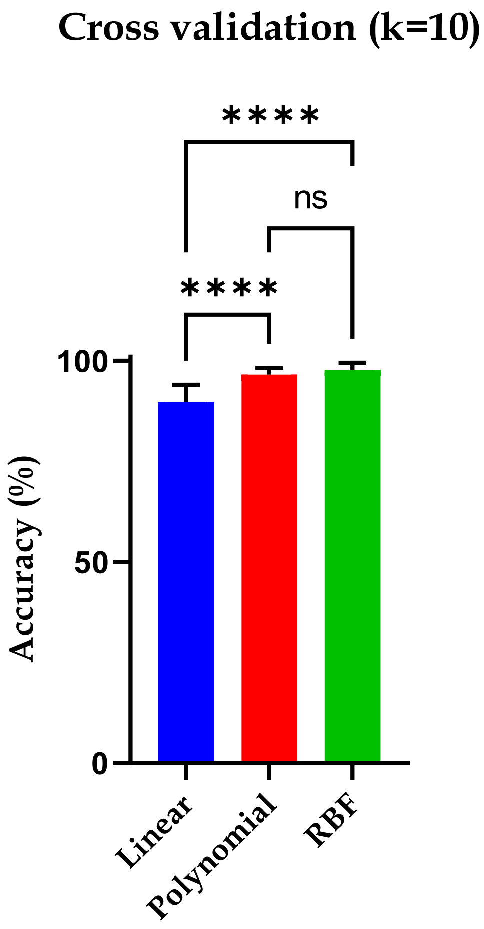

Figure S10: k-fold cross-validation (k=10) performed on the trained SVM-linear, polynomial, and RBF models (using 60% training dataset and 40% test dataset) with their mean accuracies of 89.8%, 96%, and 97.8% for SVM linear, polynomial, and RBF respectively, obtained from k-fold cross-validation data sets.

References:

(1) Priya, M.; Satish Rao, B. S.; Chandra, S.; Datta, A.; Nayak, S. G.; Mahato, K. K. Monitoring Breast Tumor Progression by Photoacoustic Measurements: A Xenograft Mice Model Study. *J Biomed Opt* **2015**, *20* (10), 105002. https://doi.org/10.1117/1.JBO.20.10.105002.

(2) Rodrigues, J.; Amin, A.; Raghushaker, C. R.; Chandra, S.; Joshi, M. B.; Prasad, K.; Rai, S.; Nayak, S. G.; Ray, S.; Mahato, K. K. Exploring Photoacoustic Spectroscopy-Based Machine Learning Together with Metabolomics to Assess Breast Tumor Progression in a Xenograft Model Ex Vivo. *Laboratory Investigation* **2021**, *101* (7), 952–965. https://doi.org/10.1038/s41374-021-00597-3.

(3) Raghushaker, C. R.; Rodrigues, J.; Nayak, S. G.; Ray, S.; Urala, A. S.; Satyamoorthy, K.; Mahato, K. K. Fluorescence and Photoacoustic Spectroscopy-Based Assessment of Mitochondrial Dysfunction in Oral Cancer Together with Machine Learning: A Pilot Study. *Anal Chem* **2021**, *93* (49), 16520–16527. https://doi.org/10.1021/acs.analchem.1c03650.

(4) Hussain, M.; Wajid, S. K.; Elzaart, A.; Berbar, M. A Comparison of SVM Kernel Functions for Breast Cancer Detection. In *2011 Eighth International Conference Computer Graphics, Imaging and Visualization*; IEEE, 2011; pp 145–150. https://doi.org/10.1109/CGIV.2011.31.
